# Hybrid Speciation and Introgression Both Underlie the Genetic Structures and Evolutionary Relationships of Three Morphologically Distinct Species of *Lilium* (Liliaceae) Forming a Hybrid Zone Along an Elevational Gradient

**DOI:** 10.3389/fpls.2020.576407

**Published:** 2020-12-07

**Authors:** Yundong Gao, AJ Harris, Huaicheng Li, Xinfen Gao

**Affiliations:** ^1^CAS Key Laboratory of Mountain Ecological Restoration and Bioresource Utilization & Ecological Restoration and Biodiversity Conservation Key Laboratory of Sichuan Province, Chengdu Institute of Biology, Chinese Academy of Sciences, Chengdu, China; ^2^Key Laboratory of Plant Resources Conservation and Sustainable Utilization, South China Botanical Garden, Chinese Academy of Sciences, Guangzhou, China; ^3^Institut für Biologie, Freie Universität Berlin, Berlin, Germany

**Keywords:** divergence with gene flow, introgression, species boundary, *Lilium*, Hengduan Mountains, Southwestern China, *Nomocharis*, hybridzation

## Abstract

We studied hybrid interactions of *Lilium meleagrinum*, *Lilium gongshanense*, and *Lilium saluenense* using an integrative approach combining population genetics, fieldwork, and phenological research. These three species occur along an elevational gradient, with *L. meleagrinum* occurring at lower elevations, *L. saluenense* at higher elevations, and *L. gongshanense* between them. The species show strong morphological differentiation despite there being no clear environmental barriers to gene flow among them. *Lilium gongshanense* is likely to have a hybrid origin based on our prior work, but its progenitors remain uncertain. We sought to determine whether gene flow occurs among these three parapatric species, and, if so, whether *L. gongshanense* is a hybrid of *L. meleagrinum* and/or *L. saluenense*. We analyzed data from multiple chloroplast genes and spacers, nuclear internal transcribed spacer (ITS), and 18 nuclear Expressed Sequence Tag-Simple Sequence Repeat (EST-SSR) microsatellites for accessions of the three species representing dense population-level sampling. We also inferred phenology by examining species in the field and using herbarium specimens. We found that there are only two types of chloroplast genomes shared among the three species and that *L. gongshanense* forms two distinct groups with closest links to other species of *Lilium* based on ITS. Taken together, *L. gongshanense* is unlikely to be a hybrid species resulting from a cross between *L. meleagrinum* and *L. saluenense*, but gene flow is occurring among the three species. The gene flow is likely to be rare according to evidence from all molecular datasets, and this is corroborated by detection of only one putative hybrid individual in the field and asynchronous phenology. We suspect that the rarity of hybridization events among the species facilitates their continued genetic separation.

## Introduction

Natural hybridization, which may lead to hybrid speciation and/or genomic reticulation, is now widely understood to be a major evolutionary mechanism in plants that can drive morphological change and adaptation, as well as shape biogeographic patterns (Soltis and Soltis, 2009). Fundamentally, hybridization can be defined as the interbreeding of individuals that are distinguishable on the basis of one or more heritable characters; often distinct species (Harrison, 1993; Harrison and Larson, 2014). Rates of hybridization are variable among kingdoms of life, but on average, around 25% of plant species are known to hybridize with at least one other species (Mallet, 2005) compared to 10% of animals. Thus, hybridization plays an important, frequent role in shaping the biodiversity of plants (Grant, 1981; Mallet, 2005; De Queiroz, 2007; Harrison and Larson, 2014; Taylor and Larson, 2019).

The potential outcomes of hybridization include hybrid speciation, introgression, reinforcement, extinction of rare species, and merger of two parental species (Abbott et al., 2013; Todesco et al., 2016). Of these, hybrid speciation and introgression via hybridization occur most frequently but are difficult to distinguish from one another using typical molecular genetics methods (Harrison and Larson, 2014; Taylor and Larson, 2019). However, methods that explicitly test rates of introgression or complex evolutionary hypotheses (e.g., Anderson and Thompson, 2002; Beerli and Palczewski, 2010; Cornuet et al., 2014) are increasingly available and help to disentangle the complicated evolutionary histories of hybridizing taxa. These methods are complemented by careful examinations of morphology (e.g., to detect the prevalence of intermediate forms) and use of ecological indicators (e.g., to determine the degree to which parental species and hybrid offspring are environmentally compatible or incompatible). Therefore, case studies, such as in ragworts (Brennan et al., 2019; Nevado et al., 2020), azaleas (Yan et al., 2019), chestnuts (Sun et al., 2020), butterflies (Capblancq et al., 2019), birds (Hooper et al., 2019), and turtles (Scott et al., 2019), continue to provide valuable insights into the complex roles of hybridization and gene flow in evolution and speciation.

The species *Lilium gongshanense* (Y.D. Gao et X.J. He) Y.D. Gao (hereafter LG) also comprises a potentially valuable model system for these kinds of complex, reticulate evolutionary processes. This species may have hybrid origins based on prior studies (Gao et al., 2012a, 2013, 2015; Gao and Gao, 2016), and it currently occurs in close proximity to two other species of *Lilium*: *L. meleagrinum* (Franchet) Y.D. Gao, and *Lilium saluenense* (Balf. f.) S.Y. Liang (hereafter LM and LS, respectively). These three species were formerly treated within the genus *Nomocharis* Franchet, which is now known to represent a polyphyletic, but morphologically cohesive, group within *Lilium* (Gao et al., 2015) comprising ca. 15 species that are endemic in eastern Asia and mostly occur at high elevations, often in alpine areas. *Nomocharis* is polyphyletic for including a monophyletic group of *Lilium* that do not share the unique morphology of *Nomocharis* but also occur at high altitudes, especially in alpine areas (i.e., non-*Nomocharis* lilies, or N-N lilies in Gao et al., 2015). Thus, although *Lilium* is a large genus of ca. 130 species, all three of these species of *Lilium*, LG, LS, and LM, are closely related to one another. In a prior study, Gao et al. (2012a) suggested that LS may be a progenitor of LG based on chloroplast DNA, while more recently, internal transcribed spacer (ITS) revealed that two distinct clades of LG that were sister to large clades of *Nomocharis* and N-N species (Gao et al., 2015).

**FIGURE 1:**
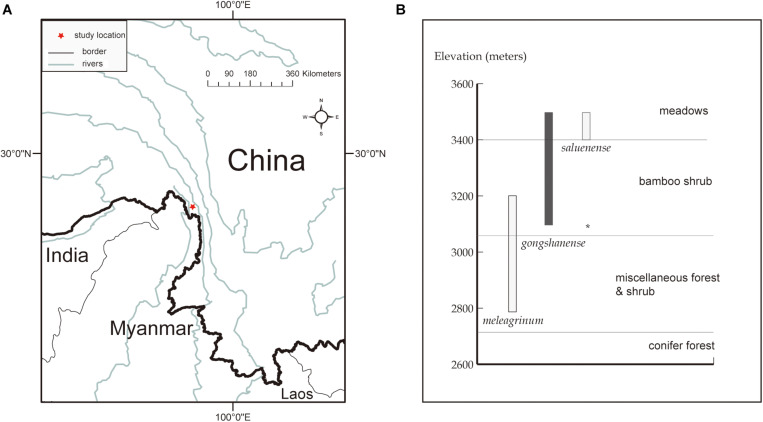
Study location and species distribution information. **(A)** The red star indicates the research spot located at northwestern Yunnan, China. **(B)** Elevational range (bars) and vegetation zones inhabited by the three *Lilium* species of present. Map generated in ESRI ArcGIS 10.0 (ESRI, 2010).

LG, LS, and LM occur parapatrically along an elevational gradient, and it remains unclear whether there is gene flow among them, especially between LG and LM and LS. All three of these focal species are endemic to China and co-occur within a small region in northwestern Yunnan Province of southwestern China (Figure 1A). Along the elevational gradient, LG occurs between LS, which grows at the highest elevations in alpine meadows, and LM, which is found among lower elevation alpine shrubs (Table 1 and Figure 1B). In the case of parapatrically distributed species, a transition zone, sometimes bearing hybrids, usually forms between them (e.g., Streisfeld and Kohn, 2005; Butlin et al., 2008; Ortiz et al., 2018), such as in the position occupied by LG. Thus, it seems plausible that LG represents a hybrid of LS and LM. However, transitional zones typically represent sharp environmental contrasts, contain few hybrids, and are generally not reported to result in hybrid speciation (e.g., Hull et al., 1996). Moreover, LG is not an intermediate of LS or LM, and we have observed few or no intermediate forms representing any of the pairs of species. All three species are morphologically distinct.

**TABLE 1:** General information of species and population composition of hybrid zone under study.

Population (size)	Species	Elevation range investigated (meters)	Plastid haploid	EST-SSR Type	Growth stage when sampling (year 2016)	Habitat
LSn*	Putative hybrid (LS × LG)	3100	H1	IV, V	Late bloom stage	Apline bamboo shrub
LSs (24)	*Lilium saluenense*	3400–3500	H1	V	In bud and vegetation phase	Alpine meadow
LNGs (25)	*Lilium gongshanense*	3300–3400	H1, H2	III	Early bloom stage	Apline bamboo shrub
LGXs (13)	*Lilium gongshanense*	3100–3300	H1	III	Full bloom stage	Alpine miscellaneous shrub
LMXs(10)	*Lilium meleagrinum*	3000–3200	H3, H5	II, IV	Full bloom stage	Alpine miscellaneous shrub
LGm(12)	*Lilium gongshanense*	3400–3500	H1	II, IV	Full bloom stage	Apline bamboo shrub
LGXn(7)	*Lilium gongshanense*	3100–3200	H1, H6	II, IV	Full bloom stage	Alpine miscellaneous shrub
LMXn(10)	*Lilium meleagrinum*	3100–3200	H3, H4, H5	II, IV	Late bloom stage	Alpine miscellaneous shrub
LMn(9)	*Lilium meleagrinum*	2800–3000	H6	I, II, III	Late bloom stage	Alpine miscellaneous shrub
LMs(25)	*Lilium meleagrinum*	2800–3000	H2, H3, H4, H5	I, III	Late bloom stage	Alpine miscellaneous shrub

*Population codes were made base on abbreviations of species names, as well as their relative distribution in the research spot (“n” as north, “s” as south and “m” for median). When two population are overlapped, a “X” symbol was applied. ^∗^LSn contains only one individual; code in bold character indicating overlapping populations (two pairs adjancently).*

**FIGURE 2:**
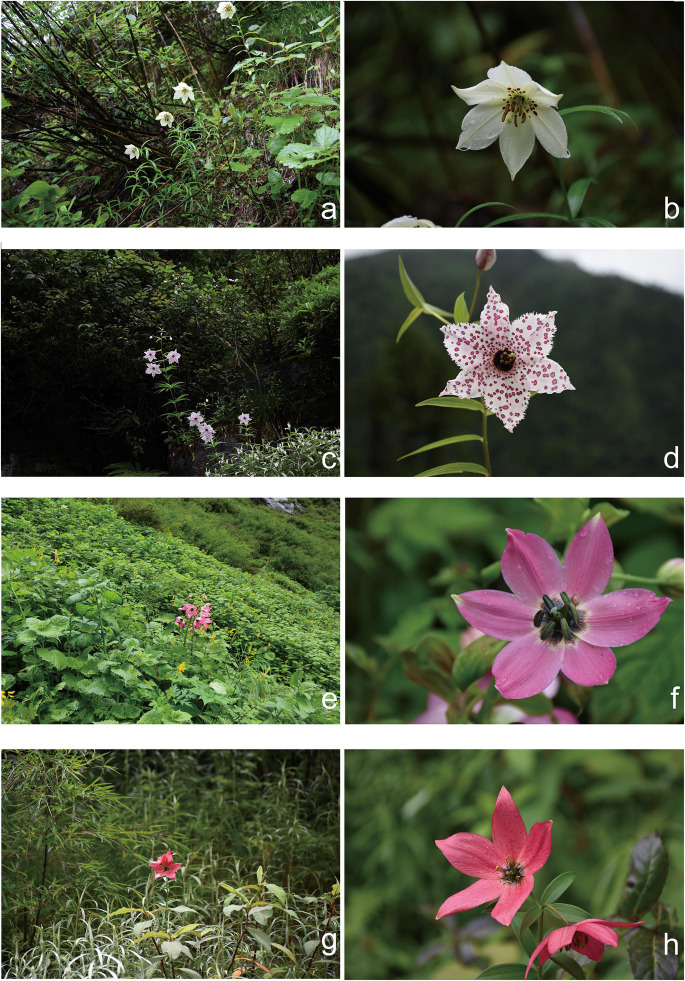
Typical habits of taxa in present study. **(a,b)** *Lilium gongshanense*; **(c,d)** *L. meleagrinum*; **(e,f)** *L. saluenense*; **(g,h)** possible F2 hybrid between *L. saluenense* and *L. gongshanense*.

The morphology of the three species has been fully discussed and illustrated in previous work (see Gao et al., 2012a, 2015; Gao and Gao, 2016). In short, LS has alternate leaves and pink tepals that have a dark purple blotch at the base and crimson blotches or spots over the rest of the tepal surfaces. LG has alternate leaves and pale yellow tepals and only few dark purple spots over the rest of the tepal surfaces. LM has opposite or whorled leaves and white or pale pink tepals. LM also differs by having fleshy, swollen, cylindrical filaments abruptly narrowing to filiform. The distinct appearances of these species make them easy to recognize in the wild (Figures 2a–f).

In this study, we sought to elucidate possible gene flow among LS, LG, and LM where they occur parapatrically and, consequently, better understand the evolutionary origins of LG. Specifically, we conducted population- and species-level analyses of these three taxa with the aim to resolve the following questions: (1) Is there ongoing gene flow among populations of LS, LG, and LM such that they form a hybrid zone or show evidence for historical gene flow? (2) If there is gene flow, do the genetic structures among them result from hybrid speciation or introgression? (3) How did the genetic structures form and how are they maintained? And (4) how do these species sustain their unique morphology if they experience gene flow? We expect that our case study of *Lilium gongshanense* can not only help to resolve the origins and evolution of the species but also help to inform increasingly more realistic approaches to integrating hybrid processes into evolutionary inference.

## Materials and Methods

### Study Location

All three focal species in this study are endemic to China and co-occur in a small area within the Gaoligong Mountain Range in northwestern Yunnan Province of southwestern China (∼N 27.80°, E98.48°, about 100 km^2^) along an elevational gradient of 2,800 to 3,500 m above sea level (Figure 1). There are limited specimen records indicating that LM and LS may also occur in adjacent areas. On the other hand, LG is only found in this area, within which we have never observed species of *Lilium* other than these three. Our study specifically focuses on a site within the Gaoligong Mountain Range that is centered around Mt. Heipu, which is divided into a northern and southern valley.

### Sampling

**FIGURE 3:**
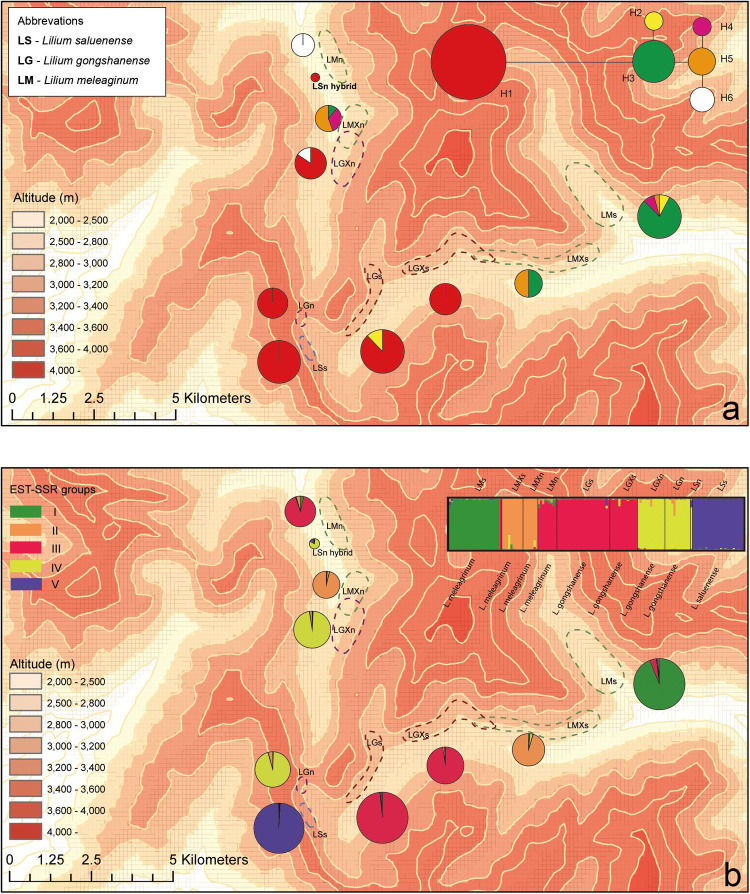
Geographic distributions of genotypes among 10 populations based on chloroplast and nuclear DNA. **(a)** Geographic distribution of six cpDNA haplotypes (H1–H6) and network of cpDNA haplotypes constructed by TCS 1.21. Sizes of circles in network are proportional to observed frequencies of haplotypes. **(b)** Geographic distribution of genotypes based on EST-SSR loci. Histogram of the STRUCTURE assignment test for the populations (139 individuals) shown at top. LM, *Lilium meleagrinum*; LG, *L. gongshanensis*; LS, *L. saluenense*. Map generated in ESRI ArcGIS 10.0 (ESRI, 2010).

In 2009, we began an investigation at the study location. Following several field seasons, we performed comprehensive, population-level sampling of species in July 2016. We sampled populations along the gradient from the peak of Mt. Heipu into both the northern and southern valleys. Within populations, our sampling of individuals comprised only leaves in order to limit the ecological impacts of our study, except that we collected one specimen per population as a voucher, which we deposited in herbarium of CDBI. The sampled leaves represented individuals growing at least 20 m apart, and we stored the leaves in silica gel at −80°C until use. We documented each sampled individual with a photograph and by obtaining geocoordinates. In total, we sampled nine populations and 139 individuals according to population size. The populations comprised LMn (*n* = 9), LMXn (*n* = 10), LGXn (*n* = 7), LGm (*n* = 12), LSs (*n* = 24, and an individual of putative hybrid origin, i.e., LSn, *n* = 1), LGs (*n* = 25), LGXs (*n* = 13), LMXs (*n* = 10), and LMs (*n* = 25) (Figure 3). The population names consist of the abbreviation of species name (LS, LG, or LM), the distribution on the northern or southern slope of Heipu (n and s), and cases where populations of two species overlap (X) (Table 1 and Figure 3).

### DNA Extraction, Sequencing, and Microsatellite Genotyping

We extracted total genomic DNA from the dried leaf tissue via a modified cetyltrimethyl ammonium bromide method (Doyle and Doyle, 1987). We amplified and sequenced ITS and the cpDNA regions *trn*L-F, *rbc*L, *mat*K, and *rpl*32-*trn*L to reconstruct phylogenetic and haplotype networks. We chose ITS because it occurs in multiple copies throughout the genome. Multiple copies may reveal a biparental genealogical signature, especially by showing two distinct copies, one from either parent. These signals can be easily be detected by observing double peaks in sequencing chromatograms. We selected the four cpDNA makers because two of them have been proposed as DNA barcodes for their high variability and amplification success (Shaw et al., 2007), and the other two showed suitable variation in prior studies of *Lilium* (Gao et al., 2013, 2015). We amplified all five markers using the primers from our previous study, Gao et al. (2015), via polymerase chain reaction (PCR) with 50 ng of genomic DNA in 20 μL reactions in a GeneAmp PCR System 9700 (Applied Biosystems, United States). We performed the ITS reactions using the following thermocycler protocol: 94°C initial denaturation and enzyme activation for 2 min; 35 cycles of 94°C denaturation for 30 s, 55°C primer annealing for 30 s, and 72°C extension for 60 s and a final extension of 72°C for 10 min. For the plastid markers, the amplification conditions were the same except that primer annealing was performed at 52°C for 45 s each cycle. We sent our amplified PCR products to Invitrogen Biotech Co., Ltd. (Shanghai, China) for purification and sequencing, which was done on an ABI-3730XL DNA sequencer. For each accession, forward and reverse sequencing reactions were performed for increased coverage.

For the resulting sequences, we performed manual editing in SeqMan Pro 7.1 (implemented in DNASTAR, Lasergene) and sequence alignment in MEGA 4 using the ClustalX method (Thompson et al., 1997) under default settings. We adjusted alignments by eye within MEGA4.0 and trimmed the alignments to the limits of the ITS and the plastid regions, respectively, by comparing with examples deposited in GenBank.

We also selected 18 primer pairs representing highly polymorphic EST-SSR markers (Supplementary Table 1) for amplification to assess demographic structures among the three species. To select the 18, we initially amplified 50 markers for a subset of individual collections of *Lilium* using primers designed for *Lilium regale* (Yuan et al., 2013), and we visualized results on a polyacrylamide gel electrophoresis. From among the 50, we selected 18 that consistently yielded good-quality amplicons. We separated PCR amplicons on a MegaBACE 1000 (GE Healthcare Biosciences, Sunnyvale, CA, United States) and scored alleles manually using GENETIC PROFILER software (version 2.2; GE Healthcare Biosciences). Final genotyping of EST-SSRs was conducted by Sangon Biotech (Shanghai) Co., Ltd. (Shanghai, China) using our amplifications.

### Population Genetic and Phylogeny Data Analyses

We first verified congruence in phylogenetic signal among the four cpDNA fragments by carrying out a partition-homogeneity test (Farris et al., 1994) using the software PAUP^∗^ 4.0b (Swofford, 2003). Based on the results, we used the combined cpDNA dataset for all subsequent analyses. For the combined cpDNA, we analyzed sequence variation in MEGA4.0 and calculated nucleotide (π) and haplotype (*h*) diversity (Nei, 1987) in DnaSP 5 (Librado and Rozas, 2009). We also compared population differentiation for phylogenetically ordered (*N*_*ST*_) and unordered (*G*_*ST*_) haplotypes and for all populations using PERMUT1.0 (Pons and Petit, 1996) and performed 1,000 permutations to determine if *N*_*ST*_ > *G*_*ST*_, which constitutes a test for phylogeographic structure. Additionally, we conducted analyses of molecular variance (AMOVA) (Excoffier et al., 1992) in the software package ARLEQUIN 3.5 (Excoffier et al., 2005) using all populations under the assumption that three species exist. We also applied AMOVA to the EST-SSR data with the same objectives, and in both cases we performed 1,023 permutations. We also investigated the phylogenetic relationships among populations represented by haplotypes using the statistical parsimony procedure for phylogenetic network estimations implemented in TCS 1.21 (Clement et al., 2000) with a 95% criterion for parsimonious connections.

To generate a phylogenetic network based on ITS, we used all new sequences generated in this study plus all available sequences of species formerly classified in *Nomocharis* and the N-N lilies (Gao et al., 2013, 2015). In total, we utilized 152 sequences of ITS, which we aligned using ClustalX in MEGA4.0 (Tamura et al., 2007) under default settings. Based on the alignment, we reconstructed an unrooted phylogenetic network in SplitsTree4 (Huson and Bryant, 2006) using the NeighborNet algorithm with Kimura 2-parameter (K2P) distances and ordinary least-squares inference of branch lengths. We performed a bootstrap analysis of the network in SplitTree4 with 1,000 replicates. We anticipated that the phylogenetic networks based on both ITS and cpDNA would help to reveal possible historical or ongoing gene flow among these three species and (in the case of ITS) their close allies that cannot be as readily detected using a bifurcating phylogenetic tree.

We primarily obtained population genetics statistics from the EST-SSR data in GenALEx version 6.501 (Peakall and Smouse, 2012) and GENEPOP version 4.0 (Raymond and Rousset, 1995) except as otherwise noted. These methods enabled us to summarize genetic structures within and among populations. In particular, we calculated differentiation between populations using *F*_*ST*_ (Weir and Cockerham, 1984), for which we determined significance at each locus and overall using 1,000 permutations. Additionally, using GenALEx, we performed a principal coordinates analyses (PCoA) to assess the clustering of genotypes.

We assessed historical gene flow based on EST-SSR datasets using MIGRATE v. 4.2.1 (Beerli and Palczewski, 2010) on CIPRES (Miller et al., 2010). Using the continuous Brownian motion model, we started five independent Markov Chain Monte Carlo (MCMC) chains, each with 100,000,000 generations and sampling every 100 steps under a constant mutation model. We discarded the first 20% of sampled generations as “burn-in.” We checked for convergence among analyses, combined the results, and obtained the mode and 95% high probability density. Based on these results, we inferred historic gene flow between each pair of species.

We also used the complete EST-SSR dataset (i.e., nine populations comprised 139 individuals, with LSn and LSs combined) to infer population structure in STRUCTURE version 2.3 (Pritchard et al., 2009). We ran STRUCTURE under the admixture model with independent allele frequencies for 100,000 MCMC generations following 10,000 burn-in generations. We performed 10 replications each in STRUCTURE for *K* = 1–20, and then we selected the optimal *K* according to Evanno et al. (2005) for downstream analyses.

We tested for specific levels of introgression among LS, LM, and LG and the hybrid status of the later using the EST-SSR markers within NewHybrids software (Anderson and Thompson, 2002) version 2.0 + Developmental^1^. NewHybrids comprises a Bayesian framework for explicitly testing hypotheses of introgression, including the existence of F1 hybrids, where the hypotheses are represented as expectations about genotype compositions. For example, F1 hybrids theoretically have 50% of loci in which allele one arises from parent one, and allele two arises from parent two. In the other 50% of loci, allele one arises from parent two, and allele two from parent one. Other levels of parental contributions to hybrids can be set as desired as well as the condition of no hybridization, and all models are evaluated simultaneously. Therefore, we tested 25 alternative hypotheses for parental contribution, ranging from none, to the existence of F1 hybrids, to very small contributions from potential donors involving only 5% of loci (Supplementary File 1). Within an analysis, hybridization can be evaluated between only two lineages or species (see also Liu et al., 2019). Thus, we performed the tests of the 25 hypotheses on three datasets: a dataset of all sampled accessions of the three species of *Lilium*, a dataset comprising only LG and LS (in total 49 individuals with population codes LGXn, LGm, LSs, including the putative hybrid LSn) (Hypothesis 1, hereafter), and a third consisting of LG and LM (in total 64 individuals with population codes LMXn, LMXs, LMn, LGs, LGXs) (Hypothesis 2, hereafter). We chose the latter two datasets based on results from analyses in STRUCTURE and our personal observations in the field. NewHybrids is performed for individuals, so we obtained average posterior probabilities for each model for each population represented within our datasets.

### Field Observations and Phenology

We visited the study location four times in 2009, 2010, 2016, and 2018. During these visits, we sought to detect any recent (i.e., in generations) hybrids, such as F1 hybrids or recent backcrosses, in the field and to observe possible mechanisms that might prevent these unique species from homogenizing. Initially, we set coarse observational transects (0.5∼1 km) and recorded the taxonomic turnover of species and habitat changes along the elevational gradient, growth stages of each population (i.e., early, full, and late bloom stages), and monitored for recent hybrids (Table 1).

Based on our initial observations, it appeared that the highest elevation species, LS, bloomed later than the other two, which largely overlapped in phenology. Thus, we compiled a robust phenological dataset for the three species using our observations at the study location and elsewhere in the Gaoligong Mountain Range and additional herbarium specimens. We realize that phenology may be strongly affected by environment, so that using samples from sites outside of the study location could introduce errors into our phenological estimations. However, all three species appear to have very narrow geographic distributions limited to a few peaks with the northern Gaoligong Range and appear to have narrow habitat (including elevation) preferences. Therefore, we expect that the additional samples are unlikely to show differing phenology based on habitat, and including them facilitated a larger sample size of these relatively rare species.

To obtain phenological information from herbarium specimens, we visited the herbaria of PE, CDBI, and KUN in China and used digital images provided online by A, E, K, and P in Europe and North America. From among available herbarium specimens, we recorded the collection dates of specimens with flowers. For the final phenological dataset, we generated boxplots and performed independent *t*-tests for flowering times using Origin 8.0 (OriginLab Corporation, United States).

### Analysis of Ecological Differences Among Species

We sought to detect ecological boundaries among these three species of *Lilium* because such boundaries may play a role in the maintenance of their unique species identities. To attempt to detect ecological boundaries, we applied PCA and ecological niche modeling (ENM) to 68 georeferenced accessions of the three species based on both our field collections and specimen records from Chinese Virtual Herbarium^2^. These comprised 20 occurrences of LG and 24 occurrences each of LS and LM. To conduct these analyses, we obtained ecological data for abiotic and biotic features of the environment from the locations of the georeferenced accessions. The abiotic environmental variables comprised all 19 temperature and precipitation variables of the bioclim dataset (Hijmans et al., 2005)^3^, elevation (Gesch et al., 1999)^4^, cloudiness (or sunshine hours per year), water limitation on plant growth (Churkina and Running, 1998)^5^,^6^ and gravel, sand, silt, and clay concentrations within top and subsoil (FAO/IIASA/ISRIC/ISSCAS/JRC, 2012). The biotic variables consisted of plant species richness by ecoregion (Kier et al., 2005)^7^, net primary productivity (Imhoff et al., 2004)^8^, percent forest and grassland cover by terrestrial ecoregion (Hoekstra et al., 2010)^9^,^10^ mammalian and bird species richness (Jenkins et al., 2013)^11^,^12^ and soil microbial diversity (Serna-Chavez et al., 2013)^13^. In all, we obtained data for 39 abiotic and biotic environmental variables. Although not all variables may be directly related to species ecological tolerances (e.g., mean annual temperature, mammalian, and bird species richness), they are well-known proxies for many aspects of the environment that are likely determinates of where species occur (Jackson et al., 2009; Hatfield et al., 2013). We resampled all variables at 30-arc-second resolution and extracted their values for the georeferenced data points using the Spatial Analyst extension of Global Information Systems (GIS) layers in ArcGIS v. 10.4.1 (ESRI, 2010).

We performed PCA analyses with all variables, which we transformed using *z* scores, for all 68 georeferenced data points. Thereafter, we used PCA axes one and two for detecting clusters of populations of the three species of *Lilium* with a k-means analysis performed using the cluster v. 2.0.8 library (Maechler et al., 2009) in R. Within k-means, we allowed up to 10 possible clusters, and we detected the optimal number of clusters by comparing each with 500 Monte Carlo simulations in the factoextra v. 1.0.5 (Kassambara and Mundt, 2017) library for R and taking the first local optimum.

We sought to utilize ENMs as multivariate models against which to test the ecological similarities among the three species of *Lilium*. Thus, we followed methods similar to Raxworthy et al. (2007). In brief, we assumed that if individuals of the same species are ecologically distinct, then models generated using individuals of one species should show a better fit when tested using randomly selected individuals of the same species compared to using individuals of a different species. Although the limitations of using coarse-scale environmental data for ecological assessments of species that occur in such close proximity is suspicious, our intention is to recover the preliminary niche difference among species and explore the key environmental variables to favor the selection of field data in the future.

Prior to ENM, we reduced the number of environmental variables by removing all but one from among those with strong covariance. To detect covariance, we generated a Euclidean distance matrix of the *z*-transformed environmental variables for the populations of *Lilium* and used that to construct a UPGMA tree of dissimilarity. Using the UPGMA, we identified clusters of variables with dissimilarities roughly in units of standard deviation on account of the *z*-transformation of the variables. Within each cluster, we retained the variable with the highest absolute value for loading on PCA axis 1. This yielded 16 variables for ENM analysis (Supplementary Figure 1).

We carefully devised a geographic extent for the ENM based on our knowledge of the geographic range of the three focal species of *Lilium* (Liang, 1995; Liang and Tamura, 2000) and of natural geological boundaries within the region. To accomplish this, we first designed a bounding box generously inclusive of the known range of LS, LG, and LM. The bounding box consisted of corners at N 31°12′34.35″, N 22°56′24.40″, E 94°13″.99″, and E 103°6″1.59″ (Supplementary Figure 2). We then obtained the geographic extent encompassing all geological provinces that contacted the bounding box and that were partially or completely terrestrial. We obtained the GIS layers representing regional geological provinces from the US Department of Interior (datasets prv3al, prv3bl, and geo8ag)^14^. The amalgamation of all geological provinces contacting the bounding box served as the geographic extent for ENM, and we clipped all environmental variables to that geography.

We performed ENM in Maxent 3.4.1 (Phillips et al., 2004; Phillips and Dudík, 2008). Within Maxent, we generated models for LS and LM and tested them using all 20 samples of *L. gongshanense*. For comparison, we also modeled each species independently using 25% subsampling and, alternatively, cross-validation for model testing. We built all models using a random seed and 20,000 points for background sampling. We made comparisons between models using the area under the curve (AUC) metric based on model testing.

### Isolation by Distance and Isolation by Environment Test

To further understand possible drivers of genetic boundaries among the three species, we calculated isolation by distance (IBD) and isolation by environment (IBE). For genetic distances, which comprised the response variable, we used pairwise *F*st values for populations resulting from the cpDNA data and Nei’s genetic distances for the nuclear EST-SSR data and performed tests using both datasets independently. For IBD, we integrated elevation into distance by using surface distances instead of standard Euclidean distances. To obtain surface distances, we converted the latitude, longitude, and elevation of our 10 sampled, georeferenced populations to *x*, *y*, and *z* coordinates representing a point on the surface of the earth within an Earth-centered, Earth-fixed Cartesian coordinate system (Swinger et al., 2003). For conversion, we used a web portal^15^ that assumed an ellipsoid earth represented by WGS 84 (Decker, 1986). Following the conversion, we generated a matrix of pairwise surface distances in R v. 3.5.3 (“Great Truth”) using the dist() function of the core library. For IBE, we obtained environmental distances by extracting environmental values for the populations representing the same variables and GIS sources used for ENM. We extracted the variables using the raster library in R (Hijmans et al., 2013) and used these data to generate a distance matrix. We generally followed Jiang et al. (2019) in transforming the predictor variable (i.e., environment in IBE and geographic distance in IBD) by the other, potentially nuisance variable (i.e., geographic distance for IBE and environment for IBD). We performed the transformation by weighting (multiplying) distances of the variable of interest by the distances of the other variable. We performed Mantel tests to assess IBE and IBD in the vegan library of R with 9,999 permutations (Jiang et al., 2019).

## Results

### Sequence Data and Tests for Gene Flow and Genetic Structures

The aligned cpDNA data represent 139 individuals from 10 populations and comprised 2,989 bp including indels or 2,965 bp when indels were excluded. The data consisted of nine single-nucleotide polymorphisms, of which all were parsimony informative. From among 139 individuals, we identified six haplotypes (H1–H6, GenBank accession numbers MN635282 to MN635305), which were divided into two major clusters by 10 steps based on single nucleotide polymorphisms (Figure 3 and Supplementary Figure 3). Among the haplotypes, H1 comprises two species (LS and LG) with six populations and 83 individuals and constituted one major cluster. The other major cluster comprised H2–H6 and included two species (LM and LG) with five populations and 56 individuals (Supplementary Figure 3). Within this cluster, we found that LG possesses only the haplotypes H2 and H6. Overall, we found that LG has two major types of chloroplast DNA and that all three species share only these two major types. These data support the existence of gene flow between LG and LM and between LG and LS.

Genetic statistics representing the cpDNA generally revealed high diversity values. In particular, the total nucleotide (π) and haplotype (*H*_*d*_) diversity across the metapopulation were 0.00113 and 0.600, respectively, and genetic diversity for the metapopulation (*H*_*T*_ = 0.673) was higher than the average diversity within populations (*H*_*S*_ = 0.206, Supplementary Table 2). Moreover, the AMOVA using cpDNA showed a very high level of variation among groups defined according to morphology and classical taxonomy (PV = 83.77%, *F*_*CT*_ = 0. 8798, *p* < 0.001, Supplementary Table 3). Taken together, these data from cpDNA support genetic distinctiveness of the three species.

The ITS sequences consisted of 152 accessions [139 newly generated and the GenBank accession numbers MN636494 to MN636632 (numbered as SUB6504140)^16^ ] including the three species of *Lilium* that are the subjects of this study, other *Lilium* formerly classified as *Nomocharis*, and the N-N lilies with accession numbers list in Supplementary Table 4. We excluded the ITS2 region of ITS because we were unable to convincingly align it during preliminary analyses due to high levels of polymorphism. The remaining characters consisted of ITS1 and 5.8S and contained 61 variable sites, of which 45 were parsimony informative.

**FIGURE 4:**
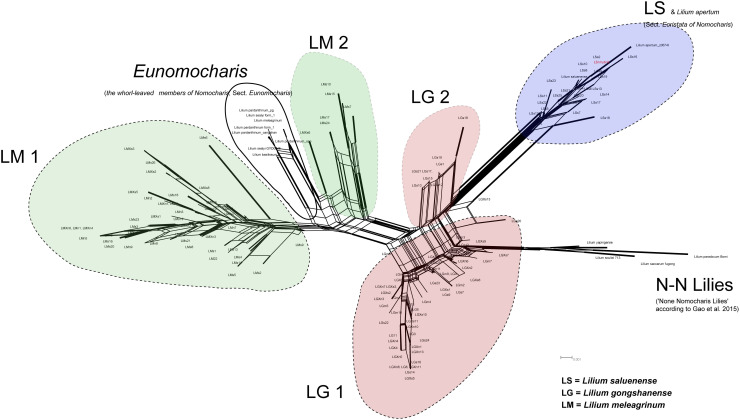
Unrooted phylogenetic network of ITS sequence conducted in SplitsTree4 (Huson and Bryant, 2006) using the NeighborNet algorithm with Kimura 2-parameter (K2P) distances and ordinary least-squares inference of branch lengths. Confidences based on bootstrap of 1,000 replicates were demonstrated by edge width.

The phylogenetic network based on nuclear ITS resolved LS with its close allies, consistent with the phylogenetic framework developed in prior studies (Gao et al., 2013, 2015). However, the species LG were poorly resolved and divided into two clusters, separated by species of N-N lilies (Gao et al., 2015), although unexpectedly, species of LM comprised two clusters separated by several former species of *Nomocharis* (Figure 4). The branch lengths for individuals of LG in the network are also much longer, on average, than for other species, suggesting high intraspecific variation (Figure 4). These data suggest that both of the parental species of LG may be N-N lilies and that it originated recently enough so that concerted evolution has not yet eliminated one parental type of ITS. More ITS sequences from other former *Nomocharis* [especially *Lilium sealyi* Y.D.Gao, *L. basilissum* (W.E. Evans) Y.D. Gao, and *L. pardanthinum* (Franchet) Y.D.Gao, formerly named *Nomocharis farreri* (W.E.Evans) Harrow, *N. basilissa* W.E.Evans and *N. pardanthina* Franchet, respectively] is needed to resolve the hybridization interactions of LM. Overall, the ITS data show that, although gene flow is occurring between LG and LM and LS, respectively, LM and LS are likely not progenitors of LG.

We detected a total of 173 alleles from among the 18 EST-SSR loci surveyed (Supplementary Table 5). We used these results and STRUCTURE to evaluate the genetic distinctiveness of the populations and three species. We tested three alternative possibilities setting structure to evaluate three different numbers of populations (*K*): (1) only two species exist, and LG is hybrid of the other two (*K* = 2); (2) there are three independent species as indicated by morphology (*K* = 3); and (3) there are five populations, which is the most optimal number according to the outcome of the statistical analysis to determine cluster number (Evanno et al., 2005) (*K* = 5, Supplementary Figure 4). When *K* = 2, LG was genetically isolated from LS (except LSn, which we believe may be a single hybrid occurrence between LG and LS; see below) but not isolated from LM (Supplementary Figure 5). At *K* = 3, all three species are resolved as genetically distinct. At *K* = 5, LG and LM were independently divided into two groups each. Comparing the results of different values of *K* (Supplementary Figure 5), it is clear that LS has greater genetic distinctiveness from LM and LG, which, together, show greater genetic associations. Moreover, the PCoA showed a clear cluster of LS and an intergrading cluster of LM and LG when plotted according to axes 1 and 2 (Supplementary Figure 6). However, the plot of PCoA axes 2 and 3 revealed two clusters of NG consistent with the ITS network analysis in SplitsTree and linked one cluster more strongly with NS. In contrast, the historic gene flow based on the EST-SSR dataset using MIGRATE showed no significant effective population exchanges among these three species (Supplementary Table 6), indicating relatively stable boundaries have existed among them.

Based on EST-SSRs, the NewHybrids analyses for Hypothesis 1 and Hypothesis 2 showed that the most probable explanation for the genotypic data was that each population consists largely of a single genetically distinct species without much hybridization (Supplementary Table 7). Under Hypothesis 1, all LS populations belonged to one genotype [0.98 posterior probability (pp)], whereas all LG populations were resolved as a second genotype (0.41–0.70 pp). Under Hypothesis 2, the LMXs and LMXn populations were resolved as belonging to one genotype (0.89 and 0.88 pp, respectively), and all other populations, including LMs, were resolved as a second genotype (0.96–0.99 pp). However, in the NewHybrids analysis with all species, all populations were resolved as having 95% of genes arising from only one species, whereas the other 5% include introgressed alleles (0.55–0.80 pp). In the absence of detecting any F1 hybrids or populations with average levels of introgression suggesting abundant F1 hybrids, hybridization events are likely to occur only rarely among the three species.

### Field Observations and Phenology

**FIGURE 5:**
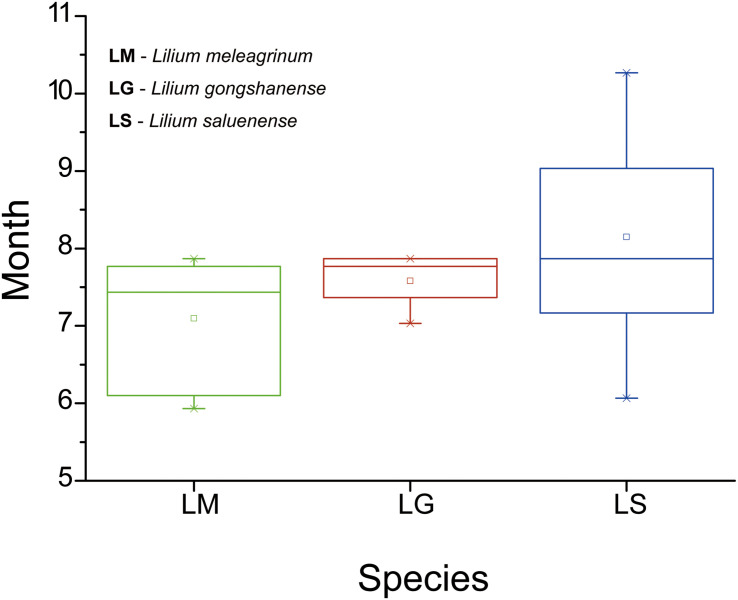
Boxplots showing differences in flowering duration of the three species based on specimen records and field observations. LM, *Lilium meleagrinum*; LG, *L. gongshanensis*; LS, *L. saluenense*.

In total (including our field observations), we collected phenological data from 44 records (LM = 15, LG = 5, LS = 24) with flowers. Our results showed that flowering time differs among the three species (Figure 5). However, only the difference between LM and LS is significant (LM vs. LS, *t* = −3.137, *p* = 0.003; LM vs. LG, *t* = −1.414, *p* = 0.174; LS vs. LG, *t* = 1.068, *p* = 0.295). The flowering time of LG overlaps partially with the flowering times of the other two species. These results suggest that, while some opportunities for gene exchange especially between LM and LG and between LS and LG exist, they may be temporally limited and therefore rare.

Via our observations of species in the field, we observed a single, isolated individual resembling LS at 3,100 m where populations of LG occur. The morphology of this individual comprised open-flat pink tepals with tiny spots, linear alternate leaves, and a dwarf habit. This individual comprised a single sample, which we treated as a distinct “population,” LSn (Figures 2g,h), in our analyses, and it was supported as being a hybrid of LG and LS based on analyses of EST-SSR in STRUCTURE (Figure 3 and Supplementary Figure 5) but not supported by NewHybrids. However, in the field, it was morphologically similar to LS and did not show intermediate features.

### Ecological Distance and Isolation

In the PCA analyses, we detected three groups of individuals using k-means (Supplementary Figure 7). These were separated on axis 1, representing primarily abiotic variables, and on axis 2, primarily consisting of biotic variables (Supplementary File 2). The three clusters did not correspond to existing species boundaries. AUCs from ENM analyses revealed that models for LS and LM were equally accurate when tested using LG as when tested using intraspecific cross-validation or subsampling (Supplementary Figure 8). The IBD tests using the cpDNA and nuclear EST-SSR showed no significant relationship between genetic and non-Euclidean geographic distance (Table 2; for the distance matrices, see Supplementary File 3). However, IBE based on each of the cpDNA and nuclear EST-SSRs showed a highly significant relationship to genetic distance. Although these species visibly occur in different environments (e.g., along an elevational gradient, within different habitats), many dimensions of the ecological niche may be shared among them, and differences in their ecological tolerances may be slight, overall.

**TABLE 2:** The results of Isolation by Distance (IBD) and Isolation by Environment (IBE) based on nuclear SSR and chloroplast (cp) datasets.

Test	Genome	Stat (Pearson’s *r*)	*p*-value
IBD	Nuclear	–0.07718	0.7118
IBD	Cp	0.1822	0.0909
IBE	Nuclear	0.789	0.0001
IBE	Cp	0.8074	0.0001

## Discussion

### Gene Flow Occurs Rarely Among the Three Species

Prior work suggested that LG is a hybrid species based on possessing two types of ITS and on morphological intermediacy (Gao et al., 2012a). Within the prior work, we inferred that the maternal progenitor of LG was LS because sampled individuals of both species shared the same chloroplast genome. However, the results here show that LG possesses two types of cpDNA genomes: one consistent with LS and another consistent with LM. Although we detected six cpDNA haplotypes (H1–H6, Figure 3 and Supplementary Figure 3), it is clear that H1 is highly distinct from the others (10 changes), whereas H2–H6 are more closely related and show only one change each. Our results suggest that both the maternal and overall history of the species is more complex than we reported in our prior study.

While the presence of gene flow among these species is supported by both the cpDNA and EST-SSRs (Supplementary Figures 3,S5), especially between LM and LG, most lines of evidence suggest that gene flow is likely rare. In the field, we observed only one individual, LSn (Figures 2g,h), which may represent an F1 hybrid or other relatively recent hybridization event. We suspect that the LSn individual represents a rare hybrid of LS and LG because of its morphology, which is LS-like, and its genetic composition according to STRUCTURE (Figure 3 and Supplementary Figure 5) and the PCoA. The STRUCTURE analysis linked LSn with LG, whereas the PCoA shows a clear relationship of this individual to LM. Taken together, this suggests that an LG individual with a background of introgression with LM hybridized with an LS individual. This is consistent with the finding from multiple lines of evidence (Figure 3b and Supplementary Figures 3,S5) that introgression plays a comparatively greater role in the evolutionary dynamic between LM and LG and also highlights the complicated nature of the evolutionary histories of these species, especially LG. Notably, NewHybrids does not support that LSn has substantial genetic contributions from either LS or LM (Supplementary Table 7; Hypothesis 1 in NewHybrids showing that LSn is a “pure” LG but with negligible support). Overall, additional field work is needed to detect what may be rare instances of hybridization facilitating gene flow and explaining introgression.

### Gene Flow Yields Biased Introgression, but Not Hybrid Speciation

Based on the ITS phylogenetic network (Figure 4), LG was divided into two major clusters representing two distinct types of ITS. Possession of two types of ITS has long been considered strong evidence for a hybrid origin, including in several studies on species of Liliaceae (e.g., in *Gagea*) (Peterson et al., 2009, 2011; Peruzzi et al., 2011). Typical patterns in the evolution of ITS have been widely studied, and normally, the multiple copies of ITS within a genome undergo concerted evolution so that all copies are inevitably identical (Zimmer et al., 1980; Koch et al., 2003). However, concerted evolution may have two outcomes in hybrid species: (1) loss of one copy and fixation of the second or (2) evolution of a new ITS type that represents a mixture of the two original ITS sequences. In cases of recent hybrid speciation, both ITS copies may be present, because concerted evolution is incomplete (reviewed in Koch et al., 2003). Therefore, in the case of an ancient hybrid speciation event, we would expect concerted evolution to have led to a single copy of ITS in the modern species. In the case of recent hybridization, the two copies of ITS should show the closest relationships with each parental species, or, as we found, with a large group of species (Figure 4), LG may result from a recent hybridization event.

Overall, our genetic results seem to suggest low, ongoing introgression between the three parapatric species under study and therefore support the existence of a hybrid zone among them. Gene flow between species comprises hybridization followed by backcrossing of the hybrid offspring into one or both parental species. Gene flow between LG and parapatric species, LM and LS, compounds the already-complex evolutionary history of the hybrid taxon. Notably, LG and LM appear to be experiencing greater gene flow than LG and LS, or, alternatively, LG and LM bear a remaining signature of ancient gene flow in addition to gene flow in the present. In either case, the outcome is that introgression between LG and LM vs. LG and LS has been asymmetrical.

### Formation and Maintenance of Genetic Structures

Our results show asymmetrical introgression between LS to LG such that backcrossing occurs more often with LG than with LS. Thus, genes of LS are more commonly introduced into populations of LG and less often the other way around. This is consistent with our observation that gene flow appears more often downslope and that there are upslope barriers (Figure 3). This observed pattern of biodiversity may result from differences in phenology in concert with dichogamy in *Lilium*, that is, the maturation of pollen before maturation of the stigma. LS has a later flowering time than either LG or LM based on our observations in the field and of museum specimens (Figure 5). Because of this, the mature pollen of LS may reach a mature stigma of LG, but pollen from LG has already dispersed by the time stigmas of LS are mature. This bias in pollination due to phenology may explain the limited gene flow we detected between LG and LS based the nuclear EST-SSRs (Supplementary Table 7, Figure 3a, and Supplementary Figure 6) showed an independent identity that LS was largely genetically distinct. In contrast with LS, LG, and LM have a more highly overlapping phenology so that interbreeding between populations of these species may be more bidirectional. Nevertheless, the asymmetry in introgression is also apparent with LG and LM, so that LM is more often the recipient of LG alleles instead of a donor to LG (Figure 3 and Supplementary Figure 5). Thus, here too, the pattern of gene flow is largely downslope, at least for the nuclear genome. More broadly, differences in phenology have also been identified as the cause of asymmetrical introgression in several genera including *Populus* (Lexer et al., 2005) and *Iris* (Martin et al., 2007). However, we still cannot exclude that other biotic factors such as pollinator interactions also affect the direction of gene flow.

Asymmetry of introgression is also apparent in the plastid genomes, such that LG and LS share more haplotypes in common than LG and LM. The plastid introgression may be best explained by seed dispersal. All species of *Lilium* produce many seeds that are encircled by flat, narrow wings and are arranged like a pile of coins inside of a capsule fruit (Liang and Tamura, 2000). Seeds of *Lilium* are thought to have very low dispersal abilities because the fruits are dry and lack animal rewards and, despite that they have wings, the seeds can only glide over short distances in most cases (Minami and Azuma, 2003; Horning and Webster, 2009). Thus, seed dispersals over larger distances, such as between populations, may occur by gravity where the capsule is the dispersal propagule for unreleased seeds. In this case, seeds of LS are likely to be distributed to populations of LG, which are downslope and situated between LS and LM, which occur further downslope.

### How Species Maintain Their Identities Despite Gene Flow

Overall, there was limited gene flow detected among species, although gene flow appears more common between LG and LM, and only low levels of introgressed nuclear genetic material are present as evidenced by our results in NewHybrids (Supplementary Table 7). Elimination of introgressed loci may occur if alleles are locally maladaptive or genetically linked with weakly deleterious alleles, e.g., as in *Heliconius* butterflies (Edelman et al., 2019). Notably, elimination of introgressed DNA from a genome takes longer when genome sizes are large (Du et al., 2017), such as in *Lilium*, which has among the largest genomes known in flowering plants [44.88–167.58 pg (Du et al., 2017); 38 ± 7.28 pg]^17^. Thus, the rather small amounts of introgressed DNA detected among the species seem to corroborate our observations in the field that hybrid speciation events are rare, perhaps even more so among LG and LS. The rarity of hybridization events between the species probably plays a crucial role in the ability of each to maintain its distinctive morphology and genetic identity (Gao et al., 2015).

One commonly invoked mechanism by which species retain their identities despite introgression is by ecological filtering, whereby the abiotic or biotic niche of a parental species may select for individuals, including hybrids, which have the traits of that parent and against the establishment and persistence of individuals with traits of the other parent or that exhibit intermediacy (Noble and Slatyer, 1980; Kraft et al., 2015). Thus, a hybrid offspring may survive and reproduce in the population of a parent with which it is morphologically similar, while intermediate prodigy or those bearing traits of the other parent may have constrained survivability and fitness. While the environmental niches of these three species are remarkably overlapping, based on our ENM results (Supplementary Figure 8), the IBE test (Table 2) using both the cpDNA and nuclear EST-SSR data shows that environment may play a significant role in maintaining species boundaries. Therefore, environmental filtering may be critical to the maintenance of these entities as distinct species.

The occurrence of parental-like hybrids and the exclusion of intermediates could be facilitated by genetic linkages on the large chromosomes of *Lilium* (Peruzzi et al., 2009; Gao et al., 2012b). Huge genome’s low recombination rate in plant also might slow the interspecific gene exchange (Tiley and Burleigh, 2015). Linkages may enable locally adaptive suites of traits that are connected to species defining morphologies to occur together much more often than we would expect for unlinked meiotic events (Rieseberg et al., 2000). Thus, environmental filtering and genetic linkage on large chromosomes may operate simultaneously to enable species to maintain unique identities. Genetic linkage merits further exploration in *Lilium*, including its relationship to ecological filtering, using whole genomic data (e.g., as in Edelman et al., 2019).

## Conclusion

Through our integrative approach using field observations, biotic and abiotic features of the environment, and population genetics based on the nuclear and chloroplast markers, we have elucidated a rather complex history for LG involving both ancient hybridization and subsequent gene flow within a hybrid zone involving species that are not progenitors. We believe this species comprises an exceptional model for assessing the robustness of methods, such as ABBA-BABA (Martin et al., 2015), which seek to infer evolutionary histories of species given confounding events. Robust methods should be able to infer multiple hybridization and introgression events at different points in the evolution of a lineage, and new models may be developed around well-understood, natural cases, such as LG. Genomic data are also needed to yield the most robust insights into the genomes involved in the origins of LG, as well as of the putative hybrid interactions of LM.

Despite the complexity of the evolutionary history of LG, we show that it is a cohesive, distinct species. Moreover, the boundaries between it and LM and LS are actively maintained, possibly by phenology and aspects of the environment, including (but likely not limited to) elevation. Boundaries between species in parapatry are maintained by intrinsic barriers to gene exchange. However, the concept of species boundary implies that species are evolutionarily independent populations that are reproductively isolated from other such species, such as according to the “biological species concept,” despite recognition of hybridization as an evolutionary process (Mallet et al., 2016). In reality, species boundaries are unlikely to be uniform in space, in time, or across the genome (Harrison and Larson, 2014; Larson et al., 2014), so that hybridization can occur while, simultaneously, the distinct character of species can be maintained. New concepts such as “genomic islands of divergence” and “divergence hitchhiking” (Bay and Ruegg, 2017; Larson et al., 2017; Ma et al., 2018) are emerging to help explain how species can sustain their identities while gene flow is active. Fundamental to these concepts is that species distinct identities may arise from selection for or against a relatively few genes and adjacent, chromosomally linked regions. The complex evolutionary history of LG makes it a suitable model for development and testing of new approaches to resolving reticulate evolutionary origins.

## Data Availability Statement

The datasets presented in this study can be found in online repositories. The names of the repository/repositories and accession number(s) can be found in the article/Supplementary Material.

## Author Contributions

YG and XG conceived and designed the experiments. HL and YG performed the experiments. YG and AH analyzed the data and contributed to the reagents, materials, analytical tools, and wrote the manuscript. All authors contributed to the article and approved the submitted version.

## Conflict of Interest

The authors declare that the research was conducted in the absence of any commercial or financial relationships that could be construed as a potential conflict of interest. The reviewer JL declared a past co-authorship with one of the authors XG to the handling editor.

## Footnotes

1 https://github.com/eriqande/newhybrids

2 http://www.cvh.ac.cn/

3 https://www.worldclim.org/

4 https://databasin.org/datasets/366a1bef53344c02bcd7d7611d5f61f7

5 https://databasin.org/datasets/6fcba40c990c483484a1d51dacf57d3c

6 https://databasin.org/datasets/321550b8dfeb459ca5856508621bb1bc

7 https://databasin.org/datasets/43478f840ac84173979b22631c2ed672

8 https://databasin.org/datasets/e416442a16ae4bb5b176515f2c0a6fff

9 https://databasin.org/datasets/ff10a966216a4995a036a2cf63f74ce2

10 https://databasin.org/datasets/735a3581a4634cc5bcc57f91f4276eb9

11 https://databasin.org/datasets/9d77254b6cea4b51b54e4e25d5f08360

12 https://databasin.org/datasets/6820867bbeee4edba14f33f75d07c629

13 https://esdac.jrc.ec.europa.eu/content/global-soil-biodiversity-maps-0

14 https://data.doi.gov/

15 https://www.oc.nps.edu/oc2902w/coord/llhxyz.htm

16 https://submit.ncbi.nlm.nih.gov/subs/genbank/SUB6504140/overview

17 https://cvalues.science.kew.org/

## Supplementary Material

Supplementary Figure 1 UPGMA tree showing similarity of *z*-transformed environmental variables among species occurrences used for ecological niche modeling. We selected one variable from each clade (black circles) with height greater than six (red line) to use in modeling. The heights are roughly in units of standard deviation due to the *z*-transformation. Within clades, we selected the variable with the highest loading on PC axis one according to our PCA analysis.

Supplementary Figure 2 Geographic extent used for modeling shown in light blue based on geological units (dark blue) contacting a bounding box generously encompassing the range of the three focal species (black) and excluding marine areas and lakes.

Supplementary Figure 3 Network of cpDNA haplotypes presented by all populations **(A)** and 3 species **(B)**, respectively. Size of circles in network are proportional to observed frequencies of haplotypes, and line distance indicated the mutations.

Supplementary Figure 4 The optimal value of *K* (*K* = 5) based on the method of Evanno et al. (2005) using EST-SSRs of STRUCTRUE analysis.

Supplementary Figure 5 Histogram of the STRUCTURE assignment test for all populations (139 individuals) inferred by eighteen nuclear microsatellite (EST-SSR) loci with different scenarios (explained in the main text).

Supplementary Figure 6 PCoA of scoring for EST-SSRs for individuals representing the three species of *Lilium*. Populations of LG are shown in brown/orange/yellow, populations of LS are shown in reds, and populations of LM are shown in blues. Percentage of variation explained is presented along with axis labels.

Supplementary Figure 7 PCA analysis of ecological boundaries among putative species of *Lilium* including three groups of individuals using k-means. Axis one representing primarily abiotic variables, and on axis two, primarily consisting of biotic variables (Supplementary File 2).

Supplementary Figure 8 Comparisons between models using the area under the curve (AUC) metric based on model testing. ENM performed in Maxent 3.4.1 (Phillips et al., 2004; Phillips and Dudík, 2008). Models generated for *Lilium saluenense* and *L. meleagrinum* and tested them using all 20 samples of *L. gongshanense*. Each species modeled independently using 25% subsampling and crossvalidation for model testing.

Supplementary Table 1 EST-SSR marker and primer sequences accordingly. Eighteen primer pairs for highly polymorphic EST-SSR for amplification to assess demographic structures among the three species were based on primers designed for *Lilium regale* (Yuan et al., 2013).

Supplementary Table 2 Genetic diversity parameters for all 10 populations of *Lilium*.

Supplementary Table 3 Hierarchical analysis of molecular variation (AMOVA) based on data from the three chloroplast DNA (cpDNA) spacers and EST-SSRs for all 9 populations of *Lilium* separated by taxa.

Supplementary Table 4 ITS accessions used in present to build the network (Figure 4).

Supplementary Table 5 Genotype data of EST-SSR including 18 locus of 139 individuals used in present. Missing data were marked as “-9” and specified in STRUCTURE analysis.

Supplementary Table 6 Posterior distribution of effective population sizes (Θ) for each species, and effective migration rates (M) between each pair of the species based on analyses in MIGRATE.

Supplementary Table 7 Table of population averages for NewHybrids models tested for all data, and two hypotheses involving a smaller number of populations. Bold type indicates the most highly supported model of parental contributions for each population.

Supplementary File 1 The 25 models tested using NewHyrbids for the full dataset as well as Hypothesis 1 and 2 (explained in text).

Supplementary File 2 Loadings of all sampled environmental variables on all axes of PCA analysis.

Supplementary File 3 Pairwise population *F*st values based on Nei genetic distance using EST-SSRs and chloroplast makers, as well as the earth surface distances instead of standard Euclidean distances used in IBD and IBE test.
