# Supplementary material for: Hybrid Speciation and Introgression Both Underlie the Genetic Structures and Evolutionary Relationships of Three Morphologically Distinct Species of Lilium (Liliaceae) Forming a Hybrid Zone Along an Elevational Gradient
Source: Front Plant Sci. 2020 Dec 7;11:576407. doi: 10.3389/fpls.2020.576407 (PMC7750405; doi:10.3389/fpls.2020.576407)
Supplement: Supplementary Table 2 — Genetic diversity parameters for all 10 populations of Lilium. [file Table_2.DOCX]

| **Table 2.** The results of Isolation by Distance (IBD) and Isolation by Environment (IBE) based on nuclear SSR and chloroplast (cp) datasets. | | | |
| --- | --- | --- | --- |
| Test | Genome | Stat (Pearson's r) | p-value |
| IBD | nuclear | -0.07718 | 0.7118 |
| IBD | cp | 0.1822 | 0.0909 |
| IBE | nuclear | 0.789 | 0.0001 |
| IBE | cp | 0.8074 | 0.0001 |
