# Supplementary material for: Hybrid Speciation and Introgression Both Underlie the Genetic Structures and Evolutionary Relationships of Three Morphologically Distinct Species of Lilium (Liliaceae) Forming a Hybrid Zone Along an Elevational Gradient
Source: Front Plant Sci. 2020 Dec 7;11:576407. doi: 10.3389/fpls.2020.576407 (PMC7750405; doi:10.3389/fpls.2020.576407)
Supplement: Supplementary Table 3 — Hierarchical analysis of molecular variation (AMOVA) based on data from the three chloroplast DNA (cpDNA) spacers and EST-SSRs for all 9 populations of Lilium separated by taxa. [file Data_Sheet_1.DOCX]

**Table S1. EST-SSR marker and primer sequences accordingly. Eighteen primer pairs for highly polymorphic EST-SSR for amplification to assess demographic structures among the three species were based on primers designed for *Lilium regale* (Yuan et al. 2013).**

| EST-SSR marker | Primer sequence **(5′ - 3′)** |
| --- | --- |
| ivflmre107 | F:TGTAACCCTTGACATAACCAT R:ATAGCCCTATATCCTGTCACT |
| ivflmre157 | F:CCCTCCATCATCTTCCTCAC R:TCTCTCCTTCCTCCCTTCAC |
| ivflmre214 | F:TGCCTCGGGATTCGGTCTTA R:TCGATGGGTAGCTGTTTCTGG |
| ivflmre265 | F:CTGGAGGGAGAAGGTGTATG R:ACAACCCAAGAACAGAAAGA |
| ivflmre350 | F:CATCCTGCTGGTCGATTTGAG R:AACGCTTGGCTCTTTGTGGC |
| ivflmre383 | F:ACGAGTCGCTGGGTTTCT R:CCGCCTTCACAGGTCAGT |
| ﻿ivflmre453 | F:AGACAAGGACCCTAACACAG R:GACAATTCCGCTGATAAAG |
| ivflmre466 | F:CTACAGGACACCCAACAAGA R:GAAGCAAGATTCCAGAGCC |
| ivflmre508 | F:GGGCCGTTTAGGGTTTCT R:CTGCCTCGTCACTGTTGG |
| ivflmre62 | F:GGTAGGAGGTTCTTCGGT R:GGCTGTTGTTGCCACTGT |
| ivflmre695 | F:GGGCTTAAGAGCTGTACATA R:GTTCTTGCTACAGAGGGAGT |
| ivflmre70 | F:CGCTTCATTCCACTCACC R:GTCTTGTCTTCCTTCGCC |
| ivflmre704 | F:ATGGAAAGCAAATGCACG R:TGAATGGGAGAAGGTTGG |
| ivflmre725 | F:TCTCCGGCATACCAAATC R:GCGTACCTGCTCCTGTTC |
| ivflmre813 | F:GTCCACAATTTGAGGCAG R:CGCTGACTGCTCTTGTGA |
| ivflmre850 | F:GCAGCGAGTGCGACAAAG R:AGATCTGGTCATCAGGAGCG |
| ivflmre957 | F:CCAACAAAAACCGAAATTAC R:CGCTGCTATGCCTAGTCAA |
| ivflmre971 | F:GGCTTGTTGTCCTTCTTG R:CCGCCACTCTTCTACCTC |

**Table S2** Genetic diversity parameters for all 10 populations of *Lilium assemblages*.

|  | | *H_S_* | *H_T_* | *N_ST_* | *G_ST_* | permutation test (*P*) |
| --- | --- | --- | --- | --- | --- | --- |
| chloroplast | 0.206 (0.0783) | | 0.673 (0.1437) | 0.880 (0.0656) | 0.695 (0.0814) | *N_ST_* > *G_ST_* (*P* <0.01) |
|  |  | |  |  |  |  |

*H_S_*, average genetic diversity within populations; *H_T_*, total genetic diversity; *N_ST_*, inter-population differentiation taking into account sequence difference; *G_ST_*, inter-population differentiation.

| **Table S3.** Hierarchical analysis of molecular variation (AMOVA) based on data from the three chloroplast DNA (cpDNA) spacers and EST-SSRs for all 9 populations of *Lilium* separated by taxa. | | | | | | |
| --- | --- | --- | --- | --- | --- | --- |
| Source of variation | d.f. | SS | VC | PV | *F*- statistics | |
| cpDNA |  |  |  |  |  | |
| Among groups | 2 | 295.09 | 3.29755 | 83.77 | FCT=0.83775* | |
| Among populations within groups | 7 | 16.803 | 0.16535 | 4.2 | FSC=0.25891** | |
| Within populations | 129 | 61.057 | 0.47331 | 12.02 | FST=0.87976** | |
| Total | 138 | 372.95 | 3.93621 |  |  | |
| EST-SSR |  |  |  |  |  | |
| Among groups | 2 | 303.607 | 1.22806 | 20.01 | FCT=0.36325** | |
| Among populations within groups | 6 | 182.925 | 1.00096 | 16.31 | FSC=0.20393** | |
| Within populations | 269 | 1051.079 | 3.90736 | 63.68 | FST=0.20013** | |
| Total | 277 | 1537.612 | 6.13638 |  |  | |
| d.f., degree of freedom; SS, sum of squares; VC, variance components; PV, percentage of variation | | | | | |  |
| **P<0.0001; *0.001<P<0.01 (based on 1023 permutations) | | | | | |  |

| Table S4. ITS accessions used in present to build the network (Fig. S4). | |  |
| --- | --- | --- |
| **Sequence ID** | **Species** | **Accession number** |
| LGs1 | *Lilium gongshanense* (Y.D.Gao & X.J.He) Y.D.Gao | MN636495 |
| LGs10 | *Lilium gongshanense* (Y.D.Gao & X.J.He) Y.D.Gao | MN636496 |
| LGs11 | *Lilium gongshanense* (Y.D.Gao & X.J.He) Y.D.Gao | MN636497 |
| LGs12 | *Lilium gongshanense* (Y.D.Gao & X.J.He) Y.D.Gao | MN636498 |
| LGs13 | *Lilium gongshanense* (Y.D.Gao & X.J.He) Y.D.Gao | MN636499 |
| LGs14 | *Lilium gongshanense* (Y.D.Gao & X.J.He) Y.D.Gao | MN636500 |
| LGs15 | *Lilium gongshanense* (Y.D.Gao & X.J.He) Y.D.Gao | MN636501 |
| LGs16 | *Lilium gongshanense* (Y.D.Gao & X.J.He) Y.D.Gao | MN636502 |
| LGs17 | *Lilium gongshanense* (Y.D.Gao & X.J.He) Y.D.Gao | MN636503 |
| LGs18 | *Lilium gongshanense* (Y.D.Gao & X.J.He) Y.D.Gao | MN636504 |
| LGs19 | *Lilium gongshanense* (Y.D.Gao & X.J.He) Y.D.Gao | MN636505 |
| LGs2 | *Lilium gongshanense* (Y.D.Gao & X.J.He) Y.D.Gao | MN636506 |
| LGs20 | *Lilium gongshanense* (Y.D.Gao & X.J.He) Y.D.Gao | MN636507 |
| LGs21 | *Lilium gongshanense* (Y.D.Gao & X.J.He) Y.D.Gao | MN636508 |
| LGs22 | *Lilium gongshanense* (Y.D.Gao & X.J.He) Y.D.Gao | MN636509 |
| LGs23 | *Lilium gongshanense* (Y.D.Gao & X.J.He) Y.D.Gao | MN636510 |
| LGs24 | *Lilium gongshanense* (Y.D.Gao & X.J.He) Y.D.Gao | MN636511 |
| LGs25 | *Lilium gongshanense* (Y.D.Gao & X.J.He) Y.D.Gao | MN636512 |
| LGs3 | *Lilium gongshanense* (Y.D.Gao & X.J.He) Y.D.Gao | MN636513 |
| LGs4 | *Lilium gongshanense* (Y.D.Gao & X.J.He) Y.D.Gao | MN636514 |
| LGs5 | *Lilium gongshanense* (Y.D.Gao & X.J.He) Y.D.Gao | MN636515 |
| LGs6 | *Lilium gongshanense* (Y.D.Gao & X.J.He) Y.D.Gao | MN636516 |
| LGs7 | *Lilium gongshanense* (Y.D.Gao & X.J.He) Y.D.Gao | MN636517 |
| LGs8 | *Lilium gongshanense* (Y.D.Gao & X.J.He) Y.D.Gao | MN636518 |
| LGs9 | *Lilium gongshanense* (Y.D.Gao & X.J.He) Y.D.Gao | MN636519 |
| LGXs1 | *Lilium gongshanense* (Y.D.Gao & X.J.He) Y.D.Gao | MN636520 |
| LGXs10 | *Lilium gongshanense* (Y.D.Gao & X.J.He) Y.D.Gao | MN636521 |
| LGXs11 | *Lilium gongshanense* (Y.D.Gao & X.J.He) Y.D.Gao | MN636522 |
| LGXs12 | *Lilium gongshanense* (Y.D.Gao & X.J.He) Y.D.Gao | MN636523 |
| LGXs13 | *Lilium gongshanense* (Y.D.Gao & X.J.He) Y.D.Gao | MN636524 |
| LGXs2 | *Lilium gongshanense* (Y.D.Gao & X.J.He) Y.D.Gao | MN636525 |
| LGXs3 | *Lilium gongshanense* (Y.D.Gao & X.J.He) Y.D.Gao | MN636526 |
| LGXs4 | *Lilium gongshanense* (Y.D.Gao & X.J.He) Y.D.Gao | MN636527 |
| LGXs5 | *Lilium gongshanense* (Y.D.Gao & X.J.He) Y.D.Gao | MN636528 |
| LGXs6 | *Lilium gongshanense* (Y.D.Gao & X.J.He) Y.D.Gao | MN636529 |
| LGXs7 | *Lilium gongshanense* (Y.D.Gao & X.J.He) Y.D.Gao | MN636530 |
| LGXs8 | *Lilium gongshanense* (Y.D.Gao & X.J.He) Y.D.Gao | MN636531 |
| LGXs9 | *Lilium gongshanense* (Y.D.Gao & X.J.He) Y.D.Gao | MN636532 |
| LGm1 | *Lilium gongshanense* (Y.D.Gao & X.J.He) Y.D.Gao | MN636533 |
| LGm10 | *Lilium gongshanense* (Y.D.Gao & X.J.He) Y.D.Gao | MN636534 |
| LGm11 | *Lilium gongshanense* (Y.D.Gao & X.J.He) Y.D.Gao | MN636535 |
| LGm12 | *Lilium gongshanense* (Y.D.Gao & X.J.He) Y.D.Gao | MN636536 |
| LGm2 | *Lilium gongshanense* (Y.D.Gao & X.J.He) Y.D.Gao | MN636537 |
| LGm3 | *Lilium gongshanense* (Y.D.Gao & X.J.He) Y.D.Gao | MN636538 |
| LGm4 | *Lilium gongshanense* (Y.D.Gao & X.J.He) Y.D.Gao | MN636539 |
| LGm5 | *Lilium gongshanense* (Y.D.Gao & X.J.He) Y.D.Gao | MN636540 |
| LGm6 | *Lilium gongshanense* (Y.D.Gao & X.J.He) Y.D.Gao | MN636541 |
| LGm7 | *Lilium gongshanense* (Y.D.Gao & X.J.He) Y.D.Gao | MN636542 |
| LGm8 | *Lilium gongshanense* (Y.D.Gao & X.J.He) Y.D.Gao | MN636543 |
| LGm9 | *Lilium gongshanense* (Y.D.Gao & X.J.He) Y.D.Gao | MN636544 |
| LMs1 | *Lilium meleagrinum* (Franchet) Y.D.Gao | MN636545 |
| LMs10 | *Lilium meleagrinum* (Franchet) Y.D.Gao | MN636546 |
| LMs11 | *Lilium meleagrinum* (Franchet) Y.D.Gao | MN636547 |
| LMs12 | *Lilium meleagrinum* (Franchet) Y.D.Gao | MN636548 |
| LMs13 | *Lilium meleagrinum* (Franchet) Y.D.Gao | MN636549 |
| LMs14 | *Lilium meleagrinum* (Franchet) Y.D.Gao | MN636550 |
| LMs15 | *Lilium meleagrinum* (Franchet) Y.D.Gao | MN636551 |
| LMs16 | *Lilium meleagrinum* (Franchet) Y.D.Gao | MN636552 |
| LMs17 | *Lilium meleagrinum* (Franchet) Y.D.Gao | MN636553 |
| LMs18 | *Lilium meleagrinum* (Franchet) Y.D.Gao | MN636554 |
| LMs19 | *Lilium meleagrinum* (Franchet) Y.D.Gao | MN636555 |
| LMs2 | *Lilium meleagrinum* (Franchet) Y.D.Gao | MN636556 |
| LMs20 | *Lilium meleagrinum* (Franchet) Y.D.Gao | MN636557 |
| LMs21 | *Lilium meleagrinum* (Franchet) Y.D.Gao | MN636558 |
| LMs22 | *Lilium meleagrinum* (Franchet) Y.D.Gao | MN636559 |
| LMs23 | *Lilium meleagrinum* (Franchet) Y.D.Gao | MN636560 |
| LMs24 | *Lilium meleagrinum* (Franchet) Y.D.Gao | MN636561 |
| LMs26 | *Lilium meleagrinum* (Franchet) Y.D.Gao | MN636562 |
| LMs3 | *Lilium meleagrinum* (Franchet) Y.D.Gao | MN636563 |
| LMs4 | *Lilium meleagrinum* (Franchet) Y.D.Gao | MN636564 |
| LMs5 | *Lilium meleagrinum* (Franchet) Y.D.Gao | MN636565 |
| LMs6 | *Lilium meleagrinum* (Franchet) Y.D.Gao | MN636566 |
| LMs7 | *Lilium meleagrinum* (Franchet) Y.D.Gao | MN636567 |
| LMs8 | *Lilium meleagrinum* (Franchet) Y.D.Gao | MN636568 |
| LMs9 | *Lilium meleagrinum* (Franchet) Y.D.Gao | MN636569 |
| LMXs1 | *Lilium meleagrinum* (Franchet) Y.D.Gao | MN636570 |
| LMXs10 | *Lilium meleagrinum* (Franchet) Y.D.Gao | MN636571 |
| LMXs2 | *Lilium meleagrinum* (Franchet) Y.D.Gao | MN636572 |
| LMXs3 | *Lilium meleagrinum* (Franchet) Y.D.Gao | MN636573 |
| LMXs5 | *Lilium meleagrinum* (Franchet) Y.D.Gao | MN636574 |
| LMXs6 | *Lilium meleagrinum* (Franchet) Y.D.Gao | MN636575 |
| LMXs7 | *Lilium meleagrinum* (Franchet) Y.D.Gao | MN636576 |
| LMXs8 | *Lilium meleagrinum* (Franchet) Y.D.Gao | MN636577 |
| LMXs9 | *Lilium meleagrinum* (Franchet) Y.D.Gao | MN636578 |
| LSs1 | *Lilium saluenense* (Balf. f.) S.Y.Liang | MN636579 |
| LSs10 | *Lilium saluenense* (Balf. f.) S.Y.Liang | MN636580 |
| LSs11 | *Lilium saluenense* (Balf. f.) S.Y.Liang | MN636581 |
| LSs12 | *Lilium saluenense* (Balf. f.) S.Y.Liang | MN636582 |
| LSs13 | *Lilium saluenense* (Balf. f.) S.Y.Liang | MN636583 |
| LSs14 | *Lilium saluenense* (Balf. f.) S.Y.Liang | MN636584 |
| LSs15 | *Lilium saluenense* (Balf. f.) S.Y.Liang | MN636585 |
| LSs16 | *Lilium saluenense* (Balf. f.) S.Y.Liang | MN636586 |
| LSs17 | *Lilium saluenense* (Balf. f.) S.Y.Liang | MN636587 |
| LSs18 | *Lilium saluenense* (Balf. f.) S.Y.Liang | MN636588 |
| LSs19 | *Lilium saluenense* (Balf. f.) S.Y.Liang | MN636589 |
| LSs2 | *Lilium saluenense* (Balf. f.) S.Y.Liang | MN636590 |
| LSs20 | *Lilium saluenense* (Balf. f.) S.Y.Liang | MN636591 |
| LSs21 | *Lilium saluenense* (Balf. f.) S.Y.Liang | MN636592 |
| LSs22 | *Lilium saluenense* (Balf. f.) S.Y.Liang | MN636593 |
| LSs23 | *Lilium saluenense* (Balf. f.) S.Y.Liang | MN636594 |
| LSs25 | *Lilium saluenense* (Balf. f.) S.Y.Liang | MN636595 |
| LSs3 | *Lilium saluenense* (Balf. f.) S.Y.Liang | MN636596 |
| LSs4 | *Lilium saluenense* (Balf. f.) S.Y.Liang | MN636597 |
| LSs5 | *Lilium saluenense* (Balf. f.) S.Y.Liang | MN636598 |
| LSs6 | *Lilium saluenense* (Balf. f.) S.Y.Liang | MN636599 |
| LSs7 | *Lilium saluenense* (Balf. f.) S.Y.Liang | MN636600 |
| LSs8 | *Lilium saluenense* (Balf. f.) S.Y.Liang | MN636601 |
| LSs9 | *Lilium saluenense* (Balf. f.) S.Y.Liang | MN636602 |
| LGXn1 | *Lilium gongshanense* (Y.D.Gao & X.J.He) Y.D.Gao | MN636603 |
| LGXn10 | *Lilium gongshanense* (Y.D.Gao & X.J.He) Y.D.Gao | MN636604 |
| LGXn11 | *Lilium gongshanense* (Y.D.Gao & X.J.He) Y.D.Gao | MN636605 |
| LGXn12 | *Lilium gongshanense* (Y.D.Gao & X.J.He) Y.D.Gao | MN636606 |
| LGXn13 | *Lilium gongshanense* (Y.D.Gao & X.J.He) Y.D.Gao | MN636607 |
| LGXn2 | *Lilium gongshanense* (Y.D.Gao & X.J.He) Y.D.Gao | MN636608 |
| LGXn3 | *Lilium gongshanense* (Y.D.Gao & X.J.He) Y.D.Gao | MN636609 |
| LGXn4 | *Lilium gongshanense* (Y.D.Gao & X.J.He) Y.D.Gao | MN636610 |
| LGXn5 | *Lilium gongshanense* (Y.D.Gao & X.J.He) Y.D.Gao | MN636611 |
| LGXn6 | *Lilium gongshanense* (Y.D.Gao & X.J.He) Y.D.Gao | MN636612 |
| LGXn7 | *Lilium gongshanense* (Y.D.Gao & X.J.He) Y.D.Gao | MN636613 |
| LGXn8 | *Lilium gongshanense* (Y.D.Gao & X.J.He) Y.D.Gao | MN636614 |
| LGXn9 | *Lilium gongshanense* (Y.D.Gao & X.J.He) Y.D.Gao | MN636615 |
| LMn1 | *Lilium meleagrinum* (Franchet) Y.D.Gao | MN636616 |
| LMn2 | *Lilium meleagrinum* (Franchet) Y.D.Gao | MN636617 |
| LMn3 | *Lilium meleagrinum* (Franchet) Y.D.Gao | MN636618 |
| LMn4 | *Lilium meleagrinum* (Franchet) Y.D.Gao | MN636619 |
| LMn5 | *Lilium meleagrinum* (Franchet) Y.D.Gao | MN636620 |
| LMn6 | *Lilium meleagrinum* (Franchet) Y.D.Gao | MN636621 |
| LMn7 | *Lilium meleagrinum* (Franchet) Y.D.Gao | MN636622 |
| LMn8 | *Lilium meleagrinum* (Franchet) Y.D.Gao | MN636623 |
| LMn9 | *Lilium meleagrinum* (Franchet) Y.D.Gao | MN636624 |
| LMXn1 | *Lilium meleagrinum* (Franchet) Y.D.Gao | MN636625 |
| LMXn2 | *Lilium meleagrinum* (Franchet) Y.D.Gao | MN636626 |
| LMXn3 | *Lilium meleagrinum* (Franchet) Y.D.Gao | MN636627 |
| LMXn4 | *Lilium meleagrinum* (Franchet) Y.D.Gao | MN636628 |
| LMXn5 | *Lilium meleagrinum* (Franchet) Y.D.Gao | MN636629 |
| LMXn6 | *Lilium meleagrinum* (Franchet) Y.D.Gao | MN636630 |
| LMXn7 | *Lilium meleagrinum* (Franchet) Y.D.Gao | MN636631 |
| LSn | *Lilium saluenense* (Balf. f.) S.Y.Liang | MN636632 |
| Lilium basilissum | *Lilium basilissum* (W.E.Evans) Y.D.Gao | HQ687260 |
| Lilium yapingense | *Lilium yapingense* Y.D. Gao | HQ687290 |
| Lilium souliei 713 | *Lilium souliei* (Franchet) Sealy | JQ724631 |
| Lilium_paradxcum_Bomi | *Lilium paradoxum* Stearn | HQ687292 |
| Lilium_saccarum_fugong | *Lilium saccatum* S. Yun Liang | HQ687291 |
| Lilium pardanthinum_pg | *Lilium pardanthinum* (Franchet) Y.D.Gao | HM045431 |
| Lilium pardanthinum form_1 | *Lilium pardanthinum* (Franchet) Y.D.Gao | JQ724635 |
| Lilium pardanthinum cangshan | *Lilium pardanthinum* (Franchet) Y.D.Gao | HM045432 |
| Lilium apertum z0674I | *Lilium apertum* Franchet | HM045433 |
| Lilium saluenense | *Lilium saluenense* (Balf. f.) S.Y.Liang | HM045434 |
| Lilium pardanthinum_spp | *Lilium pardanthinum* f. *punctulatum* Sealy | HM045435 |
| Lilium meleagrinum | *Lilium meleagrinum* (Franchet) Y.D.Gao | HM045436 |
| Lilium sealyi form_1 | *Lilium sealyi* Y.D. Gao | HM045437 |
| Lilium sealyi GYD0045_10 | *Lilium sealyi* Y.D. Gao | MN636494 |
