# Supplementary material for: Hybrid Speciation and Introgression Both Underlie the Genetic Structures and Evolutionary Relationships of Three Morphologically Distinct Species of Lilium (Liliaceae) Forming a Hybrid Zone Along an Elevational Gradient
Source: Front Plant Sci. 2020 Dec 7;11:576407. doi: 10.3389/fpls.2020.576407 (PMC7750405; doi:10.3389/fpls.2020.576407)
Supplement: Supplementary Table 6 — Posterior distribution of effective population sizes (Θ) for each species, and effective migration rates (M) between each pair of the species based on analyses in MIGRATE. [file Data_Sheet_3.DOCX]

| Table S6. Posterior distribution results of effective population sizes (Θ) of each species, and effective migration rates (M) between each pair of the species in Migrate analysis in all locus. | | | | | | | |
| --- | --- | --- | --- | --- | --- | --- | --- |
|  |  |  |  |  |  |  |  |
| **Parameter** | **2.50%** | **25.00%** | **Mode** | **75.00%** | **97.50%** | **Median** | **Mean** |
| **LS vs. LM** |  |  |  |  |  |  |  |
| Θ_NS_ | 0.0000 | 0.0000 | 0.7000 | 1.4000 | 3.4000 | 1.3667 | 0.8423 |
| Θ_NM_ | 0.8667 | 2.1333 | 3.1667 | 4.0667 | 5.4000 | 3.2333 | 3.1589 |
| M_NM_->_NS_ | 0.0000 | 0.0000 | 0.1570 | 0.3730 | 0.4070 | 2.0370 | 1.5370 |
| M_NS_->_NM_ | 0.2000 | 0.4730 | 0.6370 | 0.8000 | 1.0200 | 2.9970 | 2.0930 |
| **LS vs. LG** |  |  |  |  |  |  |  |
| Θ_NS_ | 0.0000 | 0.6667 | 1.5667 | 2.4000 | 3.6000 | 1.8333 | 1.5942 |
| Θ_NG_ | 0.0000 | 0.8000 | 1.7000 | 2.5333 | 3.6667 | 1.9000 | 1.7186 |
| M_NG_->_NS_ | 0.0730 | 0.8800 | 1.0370 | 1.1730 | 1.5000 | 0.8570 | 0.7880 |
| M_NS_->_NG_ | 0.3730 | 0.6070 | 0.7500 | 0.8930 | 1.1930 | 0.7770 | 0.7850 |
| **LM vs. LG** |  |  |  |  |  |  |  |
| Θ_NM_ | 1.5333 | 2.9333 | 3.9667 | 4.8667 | 6.3333 | 4.0333 | 4.0214 |
| Θ_NG_ | 0.6000 | 1.8000 | 2.7667 | 3.6667 | 4.8667 | 2.8333 | 2.7707 |
| M_NG_->_NM_ | 0.6670 | 0.9330 | 1.1370 | 1.3470 | 1.6470 | 1.2370 | 1.4120 |
| M_NM_->_NG_ | 0.8130 | 1.1400 | 1.3100 | 1.4870 | 1.9470 | 1.3500 | 1.4260 |
