# Supplementary material for: Hybrid Speciation and Introgression Both Underlie the Genetic Structures and Evolutionary Relationships of Three Morphologically Distinct Species of Lilium (Liliaceae) Forming a Hybrid Zone Along an Elevational Gradient
Source: Front Plant Sci. 2020 Dec 7;11:576407. doi: 10.3389/fpls.2020.576407 (PMC7750405; doi:10.3389/fpls.2020.576407)

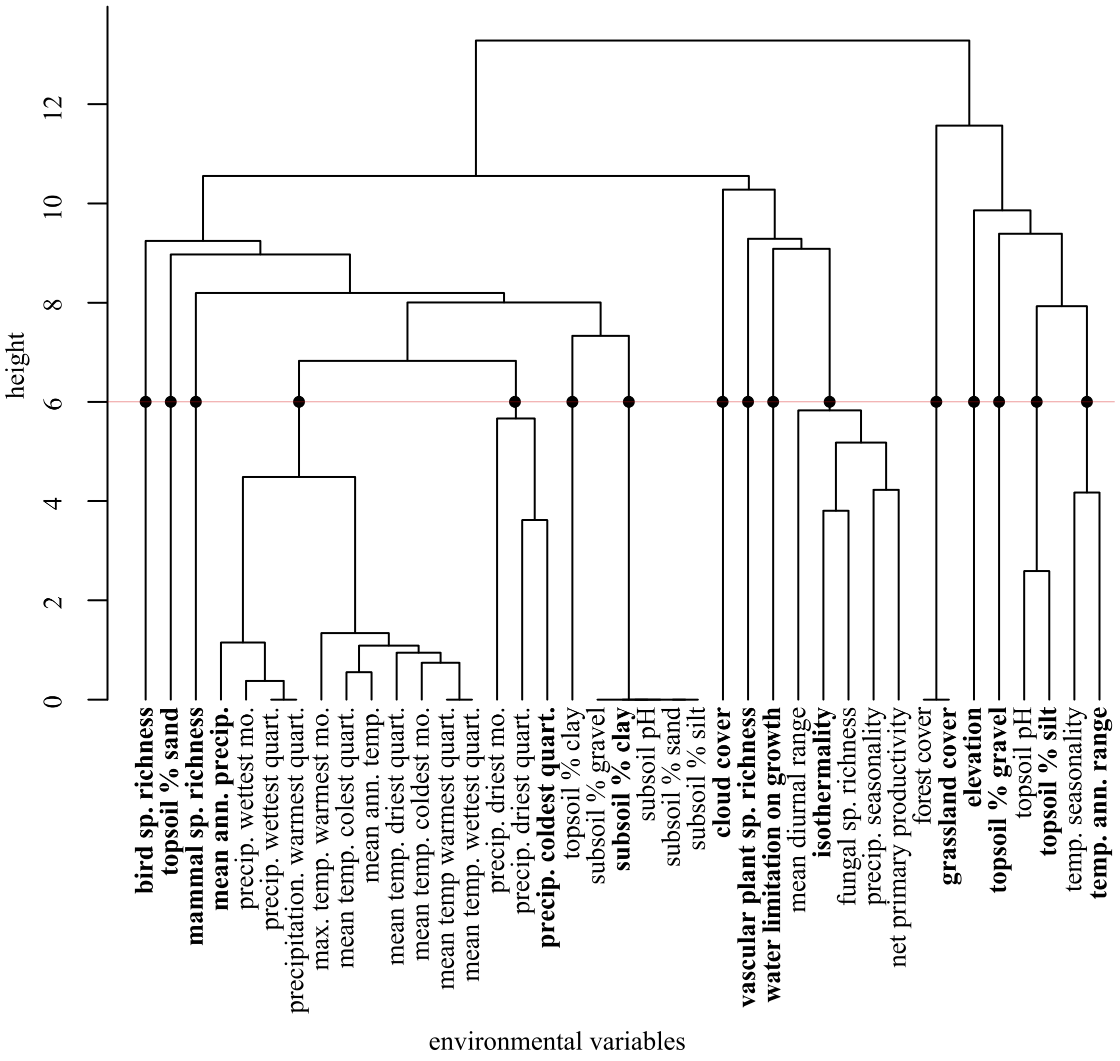

Figure S1. UPGMA tree showing similarity of z-transformed environmental variables among species occurrences used for ecological niche modeling. We selected one variable from each clade (black circles) with height greater than six (red line) to use in modeling. The heights are roughly in units of standard deviation due to the z-transformation. Within clades, we selected the variable with the highest loading on PC axis one according to our PCA analysis.

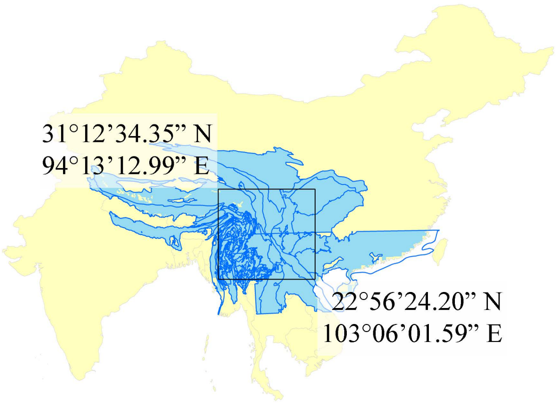

Figure S2. Geographic extent used for modeling shown in light blue based on geological units (dark blue) contacting a bounding box generously encompassing the range of the three focal species (black) and excluding marine areas and lakes.

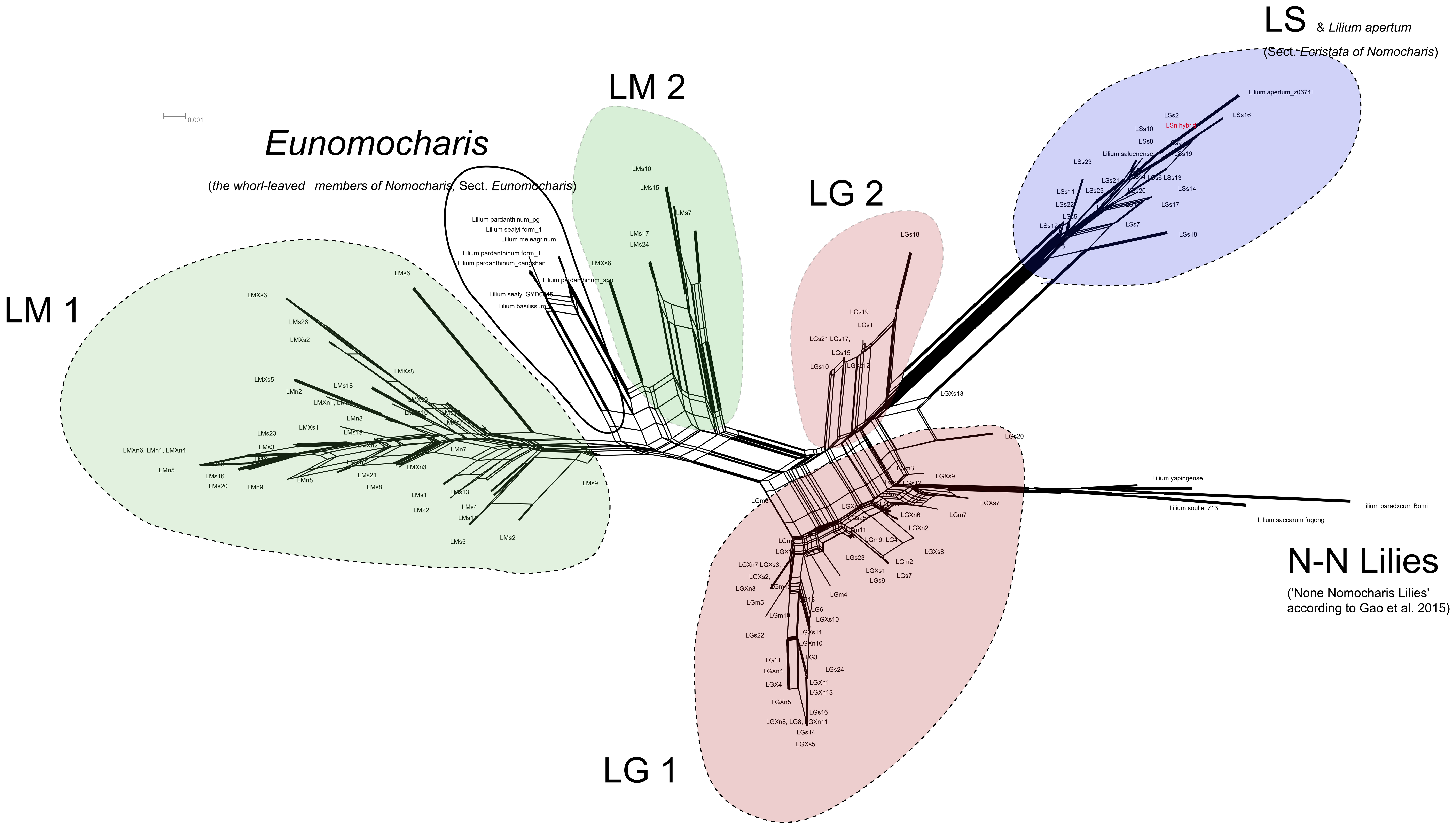

Figure S3. Unrooted phylogenetic network of ITS sequence conducted in SplitsTree4 (Huson and Bryant 2006) using the NeighborNet algorithm with Kimura 2-parameter (K2P) distances and ordinary least squares inference of branch lengths. Confidences based on bootstrap of 1000 replicates were demonstrated by edge width.

Figure S4. The optimal K value (K=5) conducted by the method of Evanno et al. (2005) using EST-SSRs of STRUCTRUE analysis.

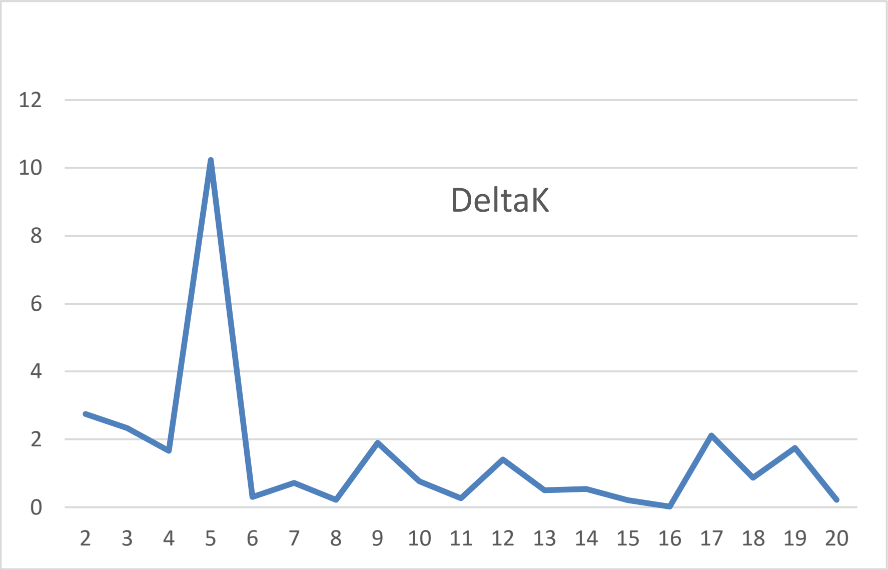

Figure S5. PCA analysis of ecological boundaries among putative species of Lilium including three groups of individuals using kmeans. Axis one representing primarily abiotic variables, and on axis two, primarily consisting of biotic variables (Supplementary file 2).

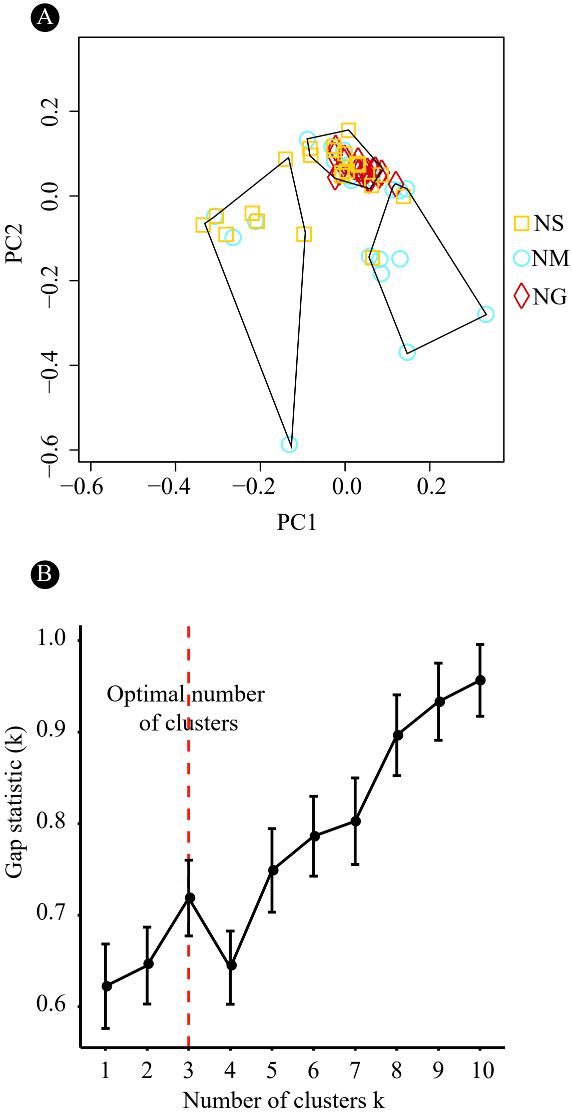

Figure S6. Comparisons between models using the area under the curve (AUC) metric based on model testing. ENM performed in Maxent 3.4.1 (Phillips et al. 2004, Phillips and Dudík 2008). Models generated for Lilium saluenensse and L. meleagrinum and tested them using all 20 samples of L. gongshanense. Each species modeled independently using 25% subsampling and crossvalidation for model testing.

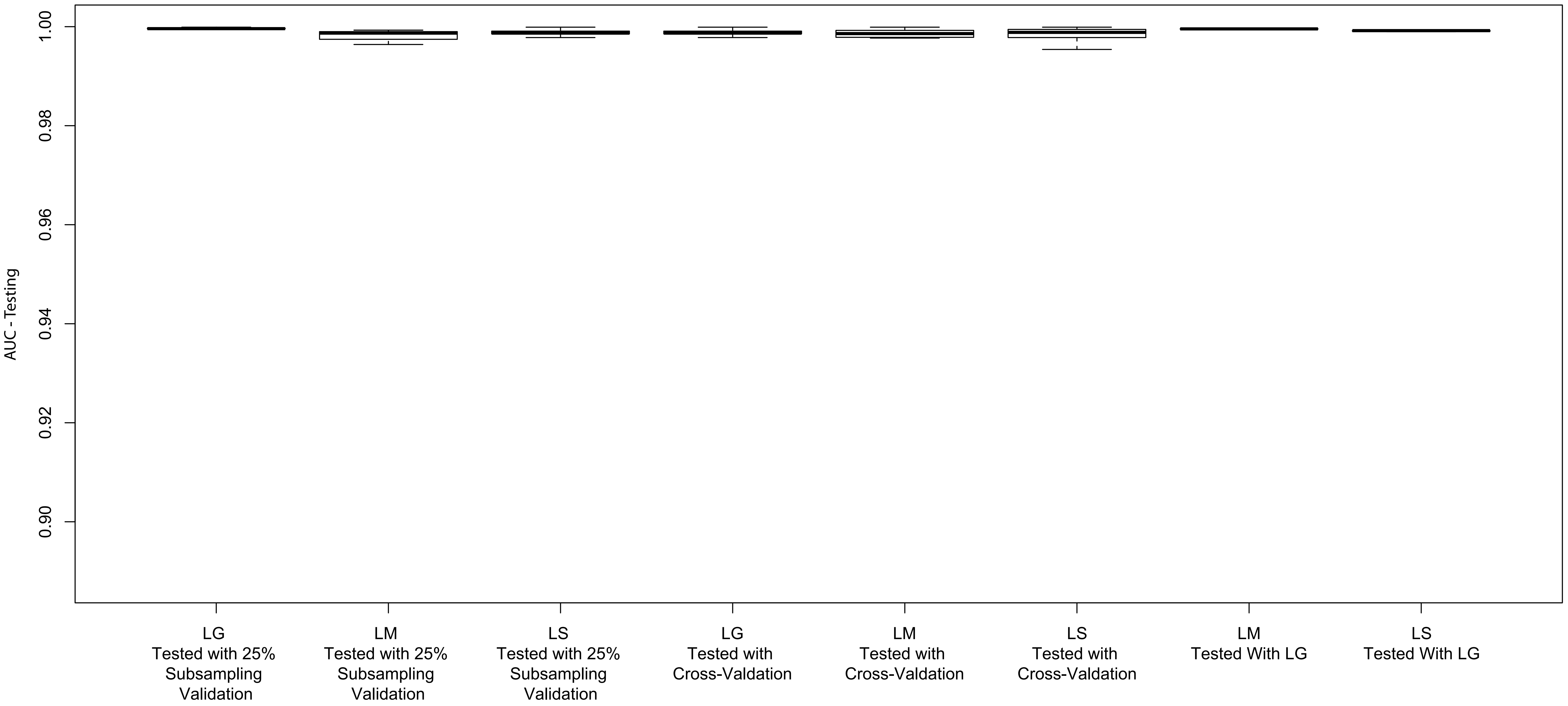

Supplement: Supplementary Figure 1 — UPGMA tree showing similarity of z-transformed environmental variables among species occurrences used for ecological niche modeling. We selected one variable from each clade (black circles) with height greater than six (red line) to use in modeling. The heights are roughly in units of standard deviation due to the z-transformation. Within clades, we selected the variable with the highest loading on PC axis one according to our PCA analysis. [file Image_1.PDF]
